# Expression of MicroRNAs in Periodontal and Peri-Implant Diseases: A Systematic Review and Meta-Analysis

**DOI:** 10.3390/ijms21114147

**Published:** 2020-06-10

**Authors:** Farah Asa’ad, Carlos Garaicoa-Pazmiño, Christer Dahlin, Lena Larsson

**Affiliations:** 1 Department of Biomaterials, Institute of Clinical Sciences, The Sahlgrenska Academy, University of Gothenburg, SE-405 30 Göteborg, Sweden; christer.dahlin@biomaterials.gu.se; 2 Department of Periodontology, Oregon Health & Science University School of Dentistry, Portland, OR 97201, USA; garaicoc@ohsu.edu; 3 Escuela de Odontologia, Universidad de Especialidades Espiritu Santo, Guayaquil 092301, Ecuador; 4 Department of ENT/Oral & Maxillofacial Surgery, NU-hospital Organization, SE-461 85 Trollhättan, Sweden; 5 Department of Periodontology, Institute of Odontology, University of Gothenburg, SE-405 30 Göteborg, Sweden; lena.larsson@odontologi.gu.se

**Keywords:** periodontitis, peri-implantitis, epigenomics, microarray analysis, real-time polymerase chain reaction, microRNAs

## Abstract

Aim: The purpose of this review was to evaluate the expression patterns of miRNAs in periodontal and peri-implant diseases, while identifying potential miRNAs with the greatest diagnostic ability as an oral fluid biomarker. Materials and methods: Human and animal studies were included when evaluating expression of miRNAs between health and different forms/stages of diseases, in which microarray and/or real-time polymerase chain reaction (RT-PCR) was carried out to detect fold changes in gene expression. After full-text analysis, 43 articles were considered for a qualitative assessment, and 16 miRNAs were selected to perform meta-analysis. Results: Based on human studies, results showed an overall upregulation of most of the evaluated miRNAs in periodontitis, with miRNA-142-3p and miRNA-146a being the most conclusive on both microarray and RT-PCR values and potentially serving as diagnostic biomarkers for disease activity. Conversely, miR-155 was the only miRNA revealing a statistically significant difference (SSD) (*p* < 0.05*) in experimental periodontitis models from RT-PCR values. Scarce scientific evidence is available from peri-implant diseases, however, most explored miRNAs in peri-implantitis were downregulated except for miR-145. Conclusions: Although our results revealed that a distinct differential expression of specific miRNAs can be noted between the state of health and disease, future research remains necessary to explore the functional role of specific miRNAs and their potential as therapeutic targets in periodontal and peri-implant diseases. MeSH Terms: periodontitis, peri-implantitis, epigenomics, microarray analysis, real-time polymerase chain reaction, microRNAs. Clinical relevance: Scientific background: Although most research identified different expression levels of miRNAs in periodontal and peri-implant diseases compared to their counterparts, their actual role in the pathogenesis of these conditions remains unclear. Therefore, we aimed to present a systematic review and meta-analysis on the expression patterns of miRNAs in periodontitis and peri-implantitis, while identifying potential miRNAs with the greatest diagnostic ability as an oral fluid biomarker. Principal findings: In periodontitis-related studies, miRNA-142-3p and miRNA-146a were the most conclusive on both microarray and RT-PCR values. Scarce scientific evidence is available from peri-implant diseases. Practical implications: Both miRNA-142-3p and miRNA-146a might serve as future diagnostic biomarkers for disease activity in periodontitis. Yet, future research remains necessary to explore the functional role of specific miRNAs and their potential as therapeutic targets in periodontal and peri-implant diseases.

## 1. Introduction

MicroRNAs (miRNAs) are a group of small noncoding RNAs of about 22 bp in length that regulate gene expression through post-transcriptional modifications by binding to the 3′-untranslated region of a target messenger RNA (mRNA) [1], which leads to suppression of gene expression either by degradation of a target mRNA or by prevention of its translation [2]. Interestingly, one miRNA can control the expression of several genes, whereas the expression of a certain gene can be controlled by several miRNAs [3].

MicroRNAs are considered as an epigenetic mechanism that modulate cellular processes, such as cell growth, apoptosis, and differentiation, and play fundamental roles in inflammatory responses and the development of diseases, e.g., cancer and rheumatoid arthritis [4].

Recently, expression of miRNAs in tissues affected by periodontitis and peri-implantitis has been investigated. Periodontitis and peri-implantitis are biofilm-induced conditions affecting teeth- and implant-supporting tissues [5], which mainly consists of Gram-negative, anaerobic, and microaerophilic bacteria that can colonize the subgingival tissues [6]. The bacterial biofilm elicits an inflammatory host response, which is influenced by environmental, genetic, and epigenetic factors [7], with the latter referring to alterations in the gene expression that are not encoded in the DNA sequence [8].

In this context, the interaction between miRNAs and *Porphyromonas gingivalis*, a key periodontal pathogen, has been extensively investigated. It was reported that miRNAs might mediate endotoxin tolerance through the modulation of mitogen-activated protein kinase (MAPK) signaling pathway, increase the sensitivity of toll-like receptors (TLRs) when exposed to bacterial lipopolysaccharide (LPS), or target the nuclear factor-kappa B (NF-κB) signaling pathway in response to bacterial stimuli [9]. Yet, the relationship between periodontal pathogens and miRNAs is still vague and requires further exploration and investigation, especially that epigenetic mechanisms have therapeutic potential for improving individualized drug therapy.

MicroRNAs have recently emerged as key regulators in bone hemostasis; they affect osteoclastogenesis either by directly regulating osteoclast activity, signaling intermediates, or through negative-feedback loops, while they control osteogenic lineage commitment of various stem cells through positive-feedback loops [10]. As such, they might mediate alveolar bone resorption, which characterizes both peri-implantitis and periodontitis [9].

Although most research identified different expression levels of miRNAs in periodontal and peri-implant diseases compared to their counterparts, their actual role in the pathogenesis of these conditions remains unclear. Conversely, miRNAs have been proposed as valuable future diagnostics tools in periodontal and peri-implant diseases as high-quality miRNAs are reportedly detectable in oral fluids [11,12,13].

Therefore, we aimed to present a systematic review and meta-analysis on the expression patterns of miRNAs in periodontitis and peri-implantitis, while identifying potential miRNAs with the greatest diagnostic ability as an oral fluid biomarker.

## 2. Results

### 2.1. Study Selection

**Figure 1:**
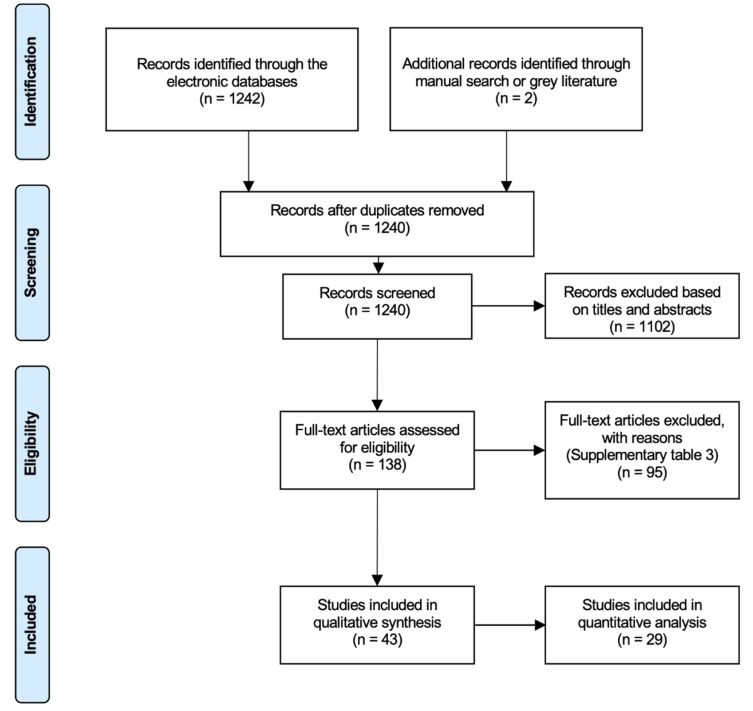
Preferred Reporting Items for Systematic Review and Meta-Analyses (PRISMA) flowchart of the screening process in the different databases.

Initial screening of electronic databases yielded a total of 1242 articles, and manual search identified 2 publications. After elimination of duplicated studies, 1240 titles and abstract were further evaluated. The screening of titles and abstracts resulted in 138 potentially relevant articles to be selected. The full text of the relevant studies was obtained and thoroughly evaluated. Next, 95 investigations were excluded due to the lack of inclusion of miRNA expression, no data on fold changes, no control group, or appropriate model as indicated in Table S3. Overall, 43 (32 human and 11 animal) studies fulfilled the inclusion and exclusion criteria and were assessed in this systematic review and meta-analysis (Figure 1).

### 2.2. Characteristics of Included Investigations

#### 2.2.1. Human Studies

The characteristics of the included human studies are presented in Table S4. It must be noted that these studies were confined to periodontal disease, as no human studies are available on miRNA and peri-implantitis. Thirty-two articles [11,12,13,14,15,16,17,18,19,20,21,22,23,24,25,26,27,28,29,30,31,32,33,34,35,36,37,38,39,40,41,42] evaluated miRNA expression in periodontitis among humans and were published from 2011 to 2020. The included studies were case–control, cross-sectional, and prospective studies, with sample sizes ranging from 3 to 150 and with an age range between 13 and 76 years.

Among these studies, 25 studies compared miRNA expression in periodontitis among healthy individuals, 3 studies were with healthy and obese, 4 studies used healthy individuals with systemic diseases, 2 studies included patients with acute coronary syndrome/coronary heart disease, while 2 studies investigated patients affected with diabetes.

The expression of miRNA was analyzed from different samples; the most harvested sample type was gingival biopsies (16 studies), followed by gingival crevicular fluid (GCF) (5 studies), periodontal ligament stem cells (PDLSC) from extracted teeth (5 studies), serum (4 studies), saliva (2 studies), and lastly, supragingival biofilm (1 study). It must be noted that in two studies, dual samples were harvested; 1 study analyzed expression from gingival biopsies and GCF, while the other study performed the analysis from serum and GCF. In all the included studies, broad and specific miRNA expression was analyzed with different techniques including RT-PCR, microarray, and next-generation sequencing.

#### 2.2.2. In Vivo (Animal) Studies

The characteristics of the included animal (in vivo) studies are presented in Table S5. A total of 11 studies were included: 9 studies [43,44,45,46,47,48,49,50,51] on the expression of miRNAs in periodontitis and 2 studies [52,53] in peri-implantitis. Regarding the animal models used, 6 studies utilized rats, 2 utilized mice, and 1 utilized monkeys for experimental periodontitis. Ligature-induced model was utilized in one study in mice and four studies in rats. Polymicrobial inoculation was utilized in one study in mice and one study in rats. One of these studies considered a dual model of ligature and polymicrobial inoculation [51]. Lastly, one experiment selected monkeys with naturally occurring periodontitis [48]. Samples were harvested from serum, gingival biopsies, and block samples (histology). The applied techniques to analyze miRNA expression were RT-PCR and microarray.

As for peri-implantitis, both studies utilized ligature-induced model in dogs. Samples were strictly harvested from gingival tissues. The techniques applied to analyze the miRNA expression were microarray and RT-PCR.

#### 2.2.3. Quality Assessment of Selected Human and Animal Studies

**Figure 2:**
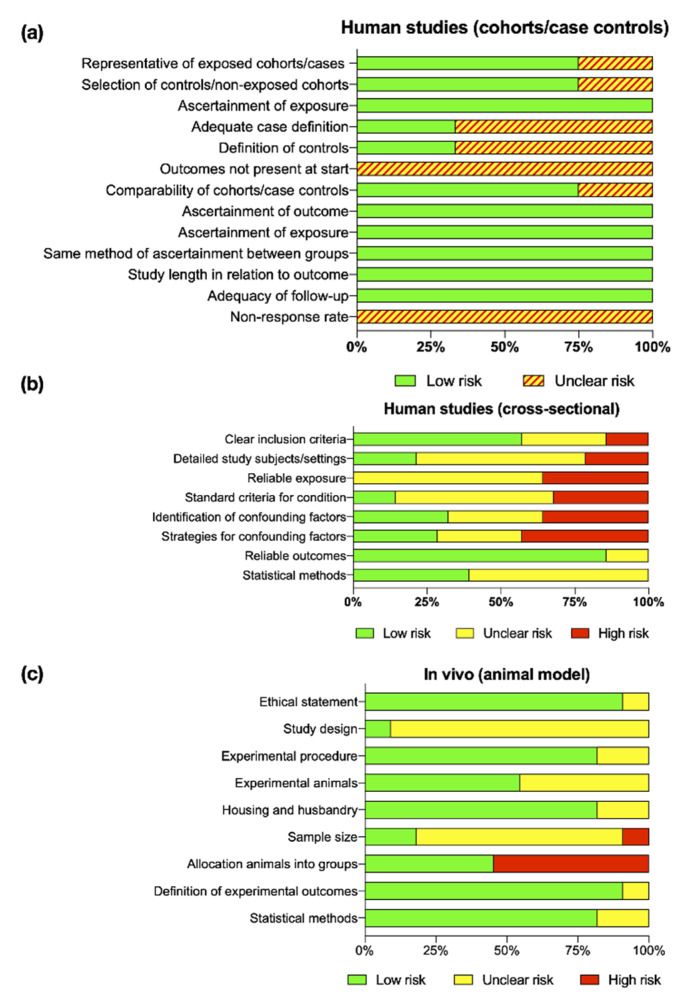
Assessment of quality and risk of bias of the included human nonrandomized cohorts/case–control (**a**), human cross-sectional (**b**), and in vivo (animal) (**c**) studies.

The quality assessment of all reviewed articles followed the Cochrane Handbook for Systematic Reviews of Interventions [54] (Tables S6–S8). Figure 2 shows the risk of bias for human nonrandomized cohorts/case–control (2a), human cross-sectional (2b), and in vivo (animal) (2c) studies. Although, the majority of human studies had clear inclusion criteria and well-accepted definitions for periodontal disease, no marginal bone levels were reported by any study and probing depths were, somehow, inconsistent with active breakdown. Therefore, the overall risk of bias was unclear for human studies. On the other hand, in vivo studies lack clinical information to determine the severity of experimental periodontitis/peri-implantitis. Nonetheless, all studies followed and complied with ethical guidelines associated with animal research and provided valid and reliable methods to assess miRNA expression. Consequently, most in vivo studies were given an unclear and low risk of bias.

### 2.3. Meta-Analysis for miRNA Expression

Among all 43 included articles, a total of 563 miRNAs were identified reporting fold changes in miRNA expression between health and periodontal/peri-implant disease (Tables S9 and S10). Due to extensiveness and limitations in the reported data, only 16 miRNAs were selected to perform a meta-analysis and were chosen if fold changes values of the same miRNA were reported in both animal and human studies or individually, in more than two and four articles from animal and human studies, respectively [13,15,17,18,20,21,22,23,26,28,29,31,32,33,35,37,38,41,42,44,45,46,47,48,49,50,51].

**Figure 3:**
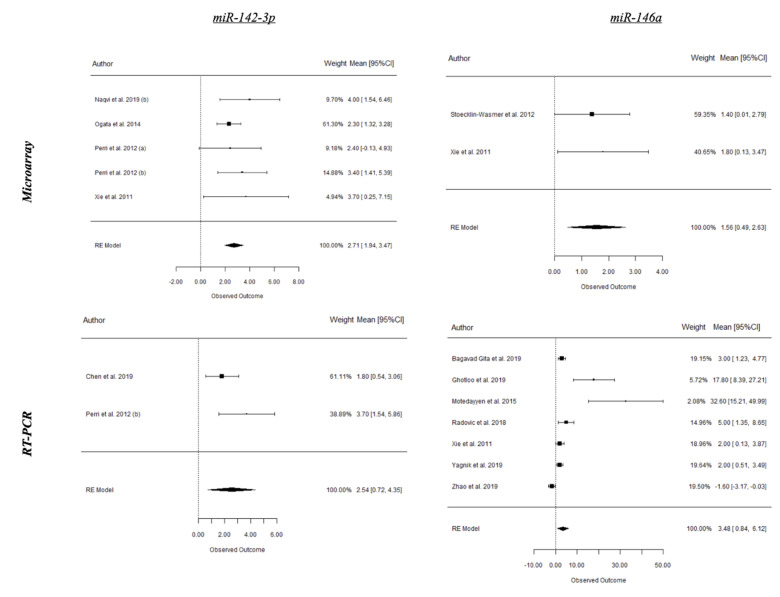
Microarray and real-time polymerase chain reaction (RT-PCR) expression of selected microRNAs (miRNAs) in periodontal health and periodontitis from human studies.

**Figure 4:**
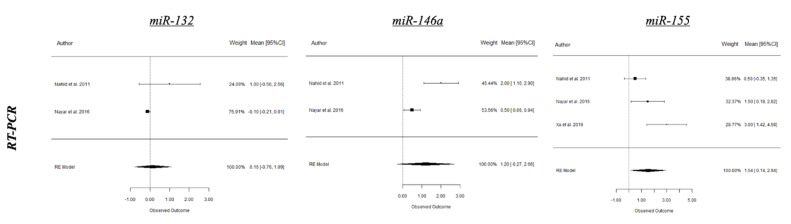
RT-PCR expression of selected miRNAs in periodontal health and experimental periodontitis.

**Figure 5:**
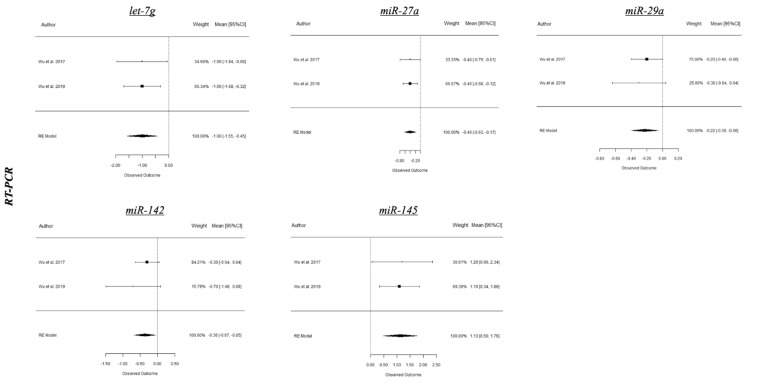
RT-PCR expression of selected miRNAs in peri-implant health and experimental peri-implantitis from animal studies.

A summary of the results from the present meta-analysis is depicted in Table 1 and forest plots of the most relevant miRNAs from periodontitis and peri-implantitis studies are shown in Figure 3, Figure 4 and Figure 5. Based on human studies, there is an overall upregulation of most of the evaluated miRNAs in periodontitis, with miR-142-3p and miR-146a being the most conclusive on both microarray and RT-PCR values and potentially serving as diagnostic biomarkers for disease activity. Conversely, miR-155 was the only miRNA revealing a statistically significant difference (SSD) (*p* < 0.05 *) in periodontitis from RT-PCR values of animal studies. Interestingly, most explored miRNAs in peri-implant disease were noted to be downregulated with an exception of miR-145 [52,53].

A statistical analysis was not possible to evaluate differences between methods of sample collection as of tissue biopsies was the procedure of choice in the majority of studies followed by serum, PDLSC from extracted teeth, block samples, saliva, GCF, and subgingival biofilm. On the other hand, a weighted mean difference (WMD) was used to compare miRNA expression differences between microarray and RT-PCR revealing that RT-PCR values are 0.71 higher than microarray and not to be SSD (*p* = 0.107, CI 95% (−0.15 to 1.58), WMD = 0.71), indicating that microarray is more sensitive to miRNA expression analysis than RT-PCR.

## 3. Discussion

MicroRNAs regulate gene expression through post-transcriptional modifications [1]. They modulate cellular functions, such as apoptosis and differentiation, and play key roles in inflammatory responses and in the development of diseases such as cancer and rheumatoid arthritis [4]. As such, expression of miRNAs has been increasingly a subject of interest in periodontitis and peri-implantitis, to better understand their role in the development of both diseases and periodontal/peri-implant tissue hemostasis.

In the present review, results from meta-analysis of human studies reflected an overall upregulation in expression of the most evaluated miRNAs in periodontitis in comparison to healthy periodontium. Interestingly, miRNA-146a was the most conclusive on both the microarray (*p* = 0.004) and RT-PCR (*p* = 0.010) values. miRNA-146a plays a critical role in the negative regulation of the innate immune response, and its dysregulation has been associated with several inflammatory disorders [26,31,38]. Regardless if it is upregulated or downregulated in periodontal disease, miRNA-146a seems to play a functional key role in periodontitis. In fact, Motedayyen et al. reported that an overexpression of miRNA-146a in patients with rapidly progressive aggressive periodontitis was accompanied by a reduction in the levels of TNF-α, IL-1β, and IL-6 [26], suggesting that the elevation of the miRNA-146a level regulates pro-inflammatory cytokines through a negative-feedback loop. Moreover, the unexpected finding of a decreased expression of these major pro-inflammatory cytokines points to the contribution of other inflammatory mediators and/or a nonimmunologic-dependent mechanism in progression of the disease.

Furthermore, miRNA-223 is significantly overexpressed in periodontitis (*p* < 0.001) and is a key regulator of osteoclastogenesis [55]. Irwandi and Vacharaksa suggested that miRNA-223 might be an important player in periodontitis-induced alveolar bone loss, having shown a consistent pattern of expression profile in gingival tissues isolated from individuals with periodontitis [56].

Other miRNAs significantly upregulated in periodontitis were miRNA-30e, miRNA-130a, miRNA-142-3p, and miRNA-210. Interestingly, these four miRNAs were reportedly overexpressed in the presence of periodontitis and obesity [31]. These specific miRNAs expression in obesity could target and post-transcriptionally modulate cytokine mRNA. In addition, they might provide new insights of how certain risk factors influence periodontal inflammation, and they are considered as novel therapeutic targets. miRNA-24-3p and miRNA-144 were also upregulated in periodontal disease, however, their role in periodontitis remains unclear, which warrants further research on these miRNAs.

Based on experimental periodontitis models, miRNA-155 expression was significantly upregulated in periodontitis (*p* = 0.031). Interestingly, conflicting findings on the expression of miRNA-155 was noted when compared to humans [33,35,37]. Such discrepancy could be as a result of differences in sample size and method applied in miRNA profiling. Since epigenetic modifications might be influenced by genetic ancestry and environmental exposure, these factors might add up to the discrepancy in results. Conversely, the impact of genetic ancestry is clearly mentioned only in one study [29], and at present, it is merely a speculation [9].

Regarding results of miRNA expression in peri-implantitis, they are confined to findings from two animal studies [52,53]. Interestingly, most miRNAs in peri-implant disease (let-7g, miRNA-27a, miRNA-29a and miRNA-142) were significantly downregulated with an exception of miRNA-145, which was significantly upregulated. In brief, Wu and colleagues demonstrated that let-7g, miRNA-27a, and miRNA-142 influenced the onset, progression, and treatment of peri-implantitis in a canine ligature-induced peri-implantitis model, highlighting the potential biological effects of the differentially expressed miRNAs and the specific enrichment of target genes involved in the MAPK signaling pathway [52,53].

It is of paramount importance to mention that several miRNAs displayed a dual regulation in advanced stages of periodontal disease. A possible explanation for this phenomenon could be due to the fact that one specific miRNA can target and regulate multiple genes [13]. Therefore, it is important to further elucidate on how the dual regulation of a specific miRNA might play a role in the progression and regression of periodontal disease.

Understanding the functional roles of miRNAs in the pathogenesis of periodontitis and peri-implantitis is very important due to their strong potential as therapeutic targets in alveolar bone regeneration. In this context, miRNAs have been shown to play an important role in the differentiation of periodontal ligament stem cells into an osteogenic lineage [25,57,58]. This could be of a great value, since stem cell therapy has reached a central role in the regenerative medicine paradigm [59]. Taking all the previous findings into consideration, it seems that miRNA therapeutics hold great promise for the future of periodontal/peri-implant therapy based on their ability to modulate the immune response to infection, either when applied in conjunction with synthetic antagomirs or by utilizing a more straightforward delivery strategy [60].

## 4. Materials and Methods

### 4.1. Information Sources

Electronic and manual literature searches were conducted by two independent reviewers (CGP and FA) in several databases, without any language restrictions, including PubMed, MEDLINE, EMBASE, and Cochrane Central Register of Controlled Trials and Cochrane Oral Health Group Trials Register databases for articles up to February 2020 published in English. The present protocol was registered in an international database of systematic reviews (PROSPERO).

### 4.2. Review (PECO) Question

The focus question for the present systematic review was developed using the PECO criteria. Do human/animal subjects (P) with periodontal/peri-implant diseases (E) compared to healthy subjects (C) have different patterns of miRNA expression (O)?

### 4.3. Search Strategy

This review was conducted according to the guidelines of the Preferred Reporting Items for Systematic Reviews and Meta-analyses (PRISMA) statement. The following search strategy was used (((“periodontal diseases” [MeSH Terms]) or periodontitis [Title/Abstract]) or peri-implantitis [Title/Abstract]) and (((“microRNA” [MeSH Terms]) or miRNA [Title/Abstract]) or mRNA [Title/Abstract]).

A second broader screening was conducted owing to the unspecific articles found indexed with the preliminary screening strategy: (((periodontal diseases [All fields]) or periodontitis [All fields]) or peri-implantitis [All fields]) and (((microRNA [All fields]) or miRNA [All fields]) or mRNA [All fields]).

Additionally, a manual search of periodontics- and implant-related journals, including Journal of Dental Research, Journal of Clinical Periodontology, Journal of Periodontology, and The International Journal of Periodontics & Restorative Dentistry, up to January 2020, was also performed to ensure a thorough screening process (CGP and FA). References of the included articles were also screened to check all the available articles. For the search of the gray literature, Google Scholar was used to identify any articles not included in the databases above.

### 4.4. Eligible Criteria

Articles were included if they evaluated the expression of miRNAs between health and different forms of periodontal (i.e., chronic or aggressive periodontitis) and peri-implant diseases (i.e., peri-implantitis) in human and animal studies. Investigations were selected if microarray and/or real-time polymerase chain reaction (RT-PCR) was carried out to detect fold changes in gene expression, of at least, one miRNA. Articles under following criteria were excluded: (I) if no fold changes were reported, (II) if no disease/experimental group was considered, (III) studies reporting on the miRNA expression from in vitro studies using cell lines not related to the oral cavity, (IV) published materials that were review articles, letters, personal opinions, book chapters, conference abstracts, and (V) articles published in a language other than English.

### 4.5. Data Extraction and Analyses

The same two authors (CGP and FA) independently reviewed all full-text articles. Discussions between the two authors initially resolved any disagreements. If the two authors did not reach an agreement, a third author (LL) made the final decision. The primary outcome considered was the fold changes of miRNA expression in periodontal/peri-implant diseases. Due to the observational nature and reported outcomes of the included investigations, only a qualitative descriptive analysis was performed upon demographic data and systematically reviewed using tables. Despite the heterogeneity among some of study subjects and subgroups, a quantitative synthesis of the included studies was possible to perform a meta-analysis. This report adhered to the PRISMA (Preferred Reporting Items for Systematic Review and Meta-Analyses) statement.

### 4.6. Risk of Bias and Quality Assessment of Selected Studies

Assessment of study quality and bias was performed for the included studies using the Cochrane review guidelines. Human nonrandomized cohorts and case–control studies were assessed using the Newcastle–Ottawa Scale (NOS) [61]. The criteria for the quality assessment of cross-sectional studies were established from the Joanna Briggs Institute (JBI) critical appraisal checklist for studies reporting prevalence data [62]. Animal research was assessed according to ARRIVE guidelines for in vivo experiments and assigned to predefined grades [63,64] (Tables S1 and S2). Two authors (CGP and FA) independently evaluated the included studies and disagreement were resolved by discussion to produce final scores. Summary assessment of risk of bias for outcomes within and across studies is represented in risk of bias graphics following Cochrane review guidelines. In summary, a low risk of bias was estimated when plausible bias is unlikely to seriously alter the results or the risk of bias was low in all key domains. The unclear risk of bias was estimated when plausible bias that raises some doubt about the results of risk of bias was in one or more key domains, whereas the high risk of bias was estimated when plausible bias that seriously weakens confidence in the results or risk of bias was in one or more key domains [54].

### 4.7. Statistical Analysis

Using a random effect model, a meta-analysis was considered to establish a statistically significant difference (*p*-values = <0.05*, <0.01**, and <0.001***) among studies reporting fold changes in miRNA expression. The DerSimonian–Liard approach was used to estimate studies variances with confidence interval (IC) of 95% and estimations were depicted using forest plots. Study heterogeneity was calculated with I^2^ and Cochran’s Q statistics, whereas publication bias was analyzed with Egger’s test and funnel plots using R 3.5.1 [65].

## 5. Study Limitations

The present systematic review was not able to establish a difference of the miRNA expression between methods for sample collection due to a high heterogeneity (i.e., biopsies, GCF, serum, and saliva), which warrants further caution when interpreting the results. On the other hand, most studies barely elucidated the functional roles of upregulated/downregulated miRNAs in the pathogenesis of periodontal and peri-implant diseases. Furthermore, due to the preliminary nature of microarray studies, further confirmation of miRNA expression in periodontitis/peri-implantitis is required.

## 6. Conclusions

We concluded that a distinct differential expression of specific miRNAs can be noted in periodontal, peri-implant disease, and healthy specimens. Moreover, miRNA expression might be affected by systemic conditions including cardiovascular diseases, obesity, and diabetes. It was noted that microarray is more sensitive method than RT-PCR for miRNA profiling, with miRNA-142-3p and miRNA-146a being the most promising miRNA biomarkers for diagnostic purposes. Scarce research was available in regard to miRNA expression and peri-implant disease. Therefore, future research is still necessary to explore the functional role of specific miRNAs and their potential role as therapeutic targets.

## Figures and Tables

**Table 1:** Summary of meta-analysis on microRNAs (miRNA) expression between microarray and real-time polymerase chain reaction (RT-PCR).

*Periodontal Health* vs. *Periodontitis (Humans)*						
miRNA	Lab Test	Fold.Dif	SE	CI 95%	*p*-Value	I^2^
miR 24-3p	Microarray	0.58	0.29	0.00 1.16	0.049*	62.0%
	RT-PCR	N/A	N/A	N/A	N/A	N/A
miR 27b-3p	Microarray	0.05	0.22	−0.37 0.48	0.803	93.1%
	RT-PCR	N/A	N/A	N/A	N/A	N/A
miR 30e	Microarray	2.34	1	0.37 4.31	0.020*	60.5%
	RT-PCR	N/A	N/A	N/A	N/A	N/A
miR 130a	Microarray	2.84	0.82	1.23 4.44	<0.001***	0.0%
	RT-PCR	N/A	N/A	N/A	N/A	N/A
miR 132	Microarray	0.75	1.22	−1.65 3.15	0.538	85.1%
	RT-PCR	N/A	N/A	N/A	N/A	N/A
miR 142-3p	Microarray	2.71	0.39	1.94 3.47	<0.001***	0.0%
	RT-PCR	2.54	0.93	0.72 4.53	0.006**	54.8%
miR 144	Microarray	1.97	0.37	1.24 2.71	<0.001***	0.0%
	RT-PCR	N/A	N/A	N/A	N/A	N/A
miR 146a	Microarray	1.56	0.54	0.49 2.63	0.004**	0.0%
	RT-PCR	3.48	1.35	0.84 6.12	0.010*	86.9%
miR 155	Microarray	−0.01	1.7	−3.34 3.32	0.995	88.1%
	RT-PCR	0.72	2.7	−4.57 6.01	0.79	91.4%
miR 210	Microarray	0.72	0.24	0.25 1.18	0.002**	9.8%
	RT-PCR	0.33	0.8	−1.23 1.89	0.681	87.9%
miR 223	Microarray	2.51	0.46	1.60 3.42	<0.001***	0.0%
	RT-PCR	4.73	3.09	−1.34 10.8	0.127	90.0%
***Periodontal health* vs. *Periodontitis (Animals)***						
**miRNA**	**Lab test**	**Fold.Dif**	**SE**	**CI 95%**	***p*-value**	**I^2^**
miR 132	Microarray	N/A	N/A	N/A	N/A	N/A
	RT-PCR	0.17	0.47	−0.76 1.09	0.726	47.70%
miR 146a	Microarray	N/A	N/A	N/A	N/A	N/A
	RT-PCR	1.2	0.75	−0.27 2.66	0.109	88.40%
miR 155	Microarray	N/A	N/A	N/A	N/A	N/A
	RT-PCR	1.54	0.71	0.14 2.94	0.031*	74.20%
***Peri-implant health* vs. *Peri-implantitis (Animals)***						
**miRNA**	**Lab test**	**Fold.Dif**	**SE**	**CI 95%**	***p*-value**	**I^2^**
let-7g	Microarray	N/A	N/A	N/A	N/A	N/A
	RT-PCR	−1	0.28	−2	<0.001***	0.00%
miR 27a	Microarray	N/A	N/A	N/A	N/A	N/A
	RT-PCR	−0.4	0.12	−0.8	<0.001***	0.00%
miR 29a	Microarray	N/A	N/A	N/A	N/A	N/A
	RT-PCR	−0.23	0.09	−0.45	0.001**	0.00%
miR 142	Microarray	N/A	N/A	N/A	N/A	N/A
	RT-PCR	−0.36	0.16	−0.72	0.022*	0.00%
miR 145	Microarray	N/A	N/A	N/A	N/A	N/A
	RT-PCR	1.13	0.32	0.50 1.76	<0.001***	0.00%

Fold.Dif: fold difference, SE: standard error, CI: confidence interval, N/A: not available.

## Supplementary Materials

Supplementary materials can be found at https://www.mdpi.com/1422-0067/21/11/4147/s1. **Table S1.** Criteria for quality assessment of animal studies using a predefined grading system (Schwarz et al. 2012) based on ARRIVE guidelines (Kilkenny et al. 2010). **Table S2.** Criteria for quality assessment of human cross-sectional studies using a predefined grading system based on The Joanna Briggs Institute (JBI) critical appraisal checklist. **Table S3.** Excluded articles. **Table S4.** Characteristics of human studies reporting miRNA expression in periodontal disease. **Table S5.** Characteristics of in vivo (animal) studies reporting miRNA expression in periodontal and peri-implant disease. **Table S6.** Quality assessment of animal research based on ARRIVE guidelines and predefined grading scores (Schwarz et al. 2012). **Table S7.** Quality assessment of nonrandomized studies based on the Newcastle–Ottawa Scale (NOS). **Table S8.** Quality assessment of human case reports, case series, and cross-sectional studies based on The Joanna Briggs Institute (JBI) critical appraisal checklist. **Table S9.** Overall miRNA fold expression changes from human studies based on microarray and RT-PCR reported values. **Table S10.** Overall miRNA fold expression changes from in vivo (animal) studies based on microarray and RT-PCR reported values.
