# Supplementary material for: Expression of MicroRNAs in Periodontal and Peri-Implant Diseases: A Systematic Review and Meta-Analysis
Source: Int J Mol Sci. 2020 Jun 10;21(11):4147. doi: 10.3390/ijms21114147 (PMC7312949; doi:10.3390/ijms21114147)
Supplement: Supplementary file 1 [file ijms-21-04147-s001.pdf]

**Supplementary Table S1. Criteria for quality assessment of animal studies using a predefined grading system (Schwarz et al. 2012) based on ARRIVE guidelines (Kilkenny et al. 2010).**

| Item | Description | Grading |
|---|---|---|
| 5 | Ethical statement – *nature of the review permission, relevant license, national and institutional guideless for the care and use of animal* | 0 = Clearly insufficient<br>1= Possible sufficient<br>2 = Clearly sufficient |
| 6 | Study design – *number of experimental and control groups, any steps to minimize bias (e.g. allocation concealment, randomization, blinding)* | 0 = Clearly insufficient<br>1= Possible sufficient<br>2 = Clearly sufficient |
| 7 | Experimental procedure – *precise details (e.g. how, when, where, why)* | 0 = Clearly insufficient<br>1= Possible sufficient<br>2 = Clearly sufficient |
| 8 | Experimental animals – *species, strain, sex, developmental stage, weight, source of animals)* | 0 = Clearly insufficient<br>1= Possible sufficient<br>2 = Clearly sufficient |
| 9 | Housing and husbandry – *conditions and welfare-related assessment and interventions* | 0 = Clearly insufficient<br>1= Possible sufficient<br>2 = Clearly sufficient |
| 10 | Sample size – *total number of animals used in each experimental group, details of calculation* | 0 = Clearly insufficient<br>1= Possible sufficient<br>2 = Clearly sufficient |
| 11 | Allocation animals to experimental groups – *randomization or matching, order in which animals were treated and assessed* | 0 = No<br>1= Yes |
| 12 | Experimental outcomes – *definition of primary and secondary outcomes* | 0 = No<br>1= Unclear/not complete<br>2 = Yes |
| 13 | Statistical methods – *details and unit of analysis* | 0 = No<br>1= Unclear/not complete<br>2 = Yes |

**Supplementary Table S2. Criteria for quality assessment of human cross-sectional studies using a predefined grading system based on The Joanna Briggs Institute (JBI) critical appraisal checklists.**

| Item | Description | Grading |
|---|---|---|
| 1 | Were the criteria for inclusion in the sample clearly defined? | 0 = No<br>1= Unclear/not complete<br>2 = Yes |
| 2 | Were the study subjects and the setting described in detail? | 0 = No<br>1= Unclear/not complete<br>2 = Yes |
| 3 | Was the exposure measured in a valid and reliable way? | 0 = No<br>1= Unclear/not complete<br>2 = Yes |
| 4 | Were objective, standard criteria used for measurement of the condition? | 0 = No<br>1= Unclear/not complete<br>2 = Yes |
| 5 | Were confounding factors identified? | 0 = No<br>1= Unclear/not complete<br>2 = Yes |
| 6 | Were strategies to deal with confounding factors stated? | 0 = No<br>1= Unclear/not complete<br>2 = Yes |
| 7 | Were the outcomes measured in a valid and reliable way? | 0 = No<br>1= Unclear/not complete<br>2 = Yes |
| 8 | Was appropriate statistical analysis used? | 0 = No<br>1= Unclear/not complete<br>2 = Yes |

**Supplementary Table S3. Excluded articles**

| Author | Reason for exclusion |
|---|---|
| Sugatani and Hruska 2007 | Explore miRNA-223 expression in mice precursor cells. No test group was reported |
| Hung et al. 2010 | Explore miRNA-146a expression in human derived PDL stem cells harvested from extracted teeth. No control group was reported |
| Schaefer et al. 2010 | Only explore the role of DEFB1 gene polymorphism and prediction of miR-expression |
| Moffatt and Lamont 2011 | Explore miRNA-200b expression in human gingival epithelial cells. No test group was reported |
| Zhou et al. 2011 | Explore miRNA expression in human PDLSC harvested from extracted teeth. No test group was reported |
| D'Aiuto & Suvan 2012 | Opinion paper |
| Li et al. 2012 | Explore miRNA expression in human derived PDL cells harvested from extracted teeth. No test group was reported |
| Honda et al. 2012 | Explore miRNA expression in human monocytes after induction of *P.g.* LPS |
| Palanisamy et al. 2012 | Literature review |
| Sato et al. 2012 | Explore polymorphism related to miRNA expression |
| Bochenek et al. 2013 | Explore lncRNA in cell lines from patients affected with agressive periodontitis |
| Cai et al. 2013 | Explore miRNA expression in human derived PDL stem cells harvested from extracted teeth. No test group was reported |
| Kadkhodazadeh et al. 2013 | Only explore the role of miRNA gene polymorphism (miR-146a and miR-499), not gene expression |
| Kagiya et al. 2013 | No experimental periodontitis was used in the animal model |
| Wallet et al. 2013 | Explore miRNA-146a expression in multiple human derived cells lines. No test group was reported |
| Xie et al. 2013 | Explore miRNA-146 expression in human gingival keratinocytes. No control group was reported |
| Neiva et al. 2014 | Only explore expression of miRNA (miR-146a and miR-155) in diabetes-free and diabetes patients, not periodontitis. |
| Ouhara et al. 2014 | Explore miRNA-584 expression in human gingival keratinocytes. No test group was reported |
| Fordham et al. 2015 | Explore miRNA expression in serum-derived human monocytes |
| Jiang et al. 2015 | Explore miRNA-146a expression in human derived PDL cells harvested from extracted teeth. No test group was reported |
| Kebschull & Papapanou 2015 | Literature review |
| Kim et al. 2015 | Literature review |
| Marques-Rocha et al. 2015 | Literature review |
| Meyle et al. 2015 | Literature review |
| Ng et al. 2015 | Only explore expression of miRNA (miR-18b and miR-1305) in smokers and non-smokers patients, not periodontitis. |
| Zhang et al. 2015 | Compared expression profiles between serum and saliva from patients affected by periodontal disease (chronic vs refractory) but looking at expressed genes and not expression levels of miRNAs |
| Zhang et al. 2015 | Explore role of Rhizoma Dioscoreae extract to prevent bone loss in ovariectomized rats. No experimental model was used. |
| Zou et al. 2015 | Explore lncRNA expression in patients affected with periodontitis |
| Chen et al. 2016 | Explore miRNA expression in human gingival keratinocytes. No test group was reported |
| Du et al. 2016 | Explore miRNA expression in human derived PDL stem cells harvested from extracted teeth. No test group was reported |

| | |
|---|---|
| Hong et al. 2016 | Explore miRNA expression in human derived bone marrow MSC and PDL fibroblasts harvested from extracted teeth. No test group was reported |
| Ibrahim et al. 2016 | No experimental periodontitis was used in the animal model |
| Irwandi & Vacharaksa 2016 | Literature review |
| Kagiya 2016 | Literature review |
| Miller et al. 2016 | No experimental periodontitis was used in the animal model |
| Park et al. 2016 | Explore miRNA expression in serum-derived human monocytes and periodontal pathogens. No test group was reported |
| Schmalz et al. 2016 | Systematic review |
| Venugopal et al. 2016 | Only explore the role of miRNA gene polymorphism (miR-146a and miR-196a2), not gene expression |
| Wang et al. 2016 | Primarily explored lncRNAs and interaction with miR-182 by using PDL stem cells from patients affected by periodontal disease. Also, explored lncRNA and interaction between miR-182 in an animal model without periodontitis. |
| Zanetti el al. 2016 | Explore miRNA expression in human oral epithelial cells. No test group was reported |
| Cao et al. 2017 | Explore miRNA-214 expression in human derived PDL stem cells harvested from extracted teeth. No test group was reported |
| Chen and Lui 2017 | Explore miRNA-1305 expression in human derived PDL stem cells harvested from extracted teeth. No test group was reported |
| Choi et al. 2017 | Explore miRNA expression only from periodontal pathogenes |
| de Rei et al. 2017 | Literature review |
| Gu et al. 2017 | Explore miRNA expression in human derived PDL stem cells harvested from extracted teeth. No test group was reported |
| Luan et al. 2017 | Literature review |
| Li et al. 2017 | Explore miRNA-142 in serum samples from patients affected with chronic periodontitis. No control group and no fold change was reported |
| Menini et al. 2017 | Only explore expression of miRNA expression between implants with different surfaces, not peri-implantitis. |
| Olsen et al. 2017 | Literature review |
| Sehic et al. 2017 | Literature review |
| Self-Fordham et al. 2017 | Literature review |
| Venugopal et al. 2017 | Only explore the role of miRNA gene polymorphism (miR-125a and miR-499a), not gene expression |
| Wang et al. 2017 | Explore miRNA-155-3p expression in mouse cementoblasts. No test group was reported |
| Wu et al. 2017 | Explore miRNA-126 expression in human gingival fibroblasts from gingival biopsies. No test group was reported |
| Yan et al. 2017 | Explore miRNA-22 expression in human derived PDL stem cells harvested from extracted teeth. No test group was reported |
| Yang et al. 2017 | Explore miRNA-21 expression in human derived PDL stem cells harvested from extracted teeth of healthy and periodontitis affected patients. No fold changes were reported |
| Yao et al. 2017 | Explore miRNA-214 expression in human derived PDL cells harvested from extracted teeth. No test group was reported |
| Bourbour et al. 2018 | Literature review |
| Ge et al. 2018 | Explore miRNA-543 expression in human derived PDL stem cells harvested from extracted teeth. No test group was reported |
| Irwandi et al. 2018 | Explore miRNA-302-3p expression in human derived osteblast-like cells harvested from mandible during third molar extraction. No test group was reported |
| Jiang et al. 2018 | No experimental periodontitis was used in the animal model |
| Li et al. 2018b | Compared miRNA expression between chronic periodontitis and oral squamous cell carcinoma. No control group (healthy gingiva) |

| | |
|---|---|
| Li et al. 2018c | Explore miRNA expression in healthy and periodontitis affected patients. No fold changes were reported |
| Liu et al. 2018 | Use nanofibrous spongy microspheres to release miR-10a and other growth factors to prevent/reduce bone loss in a ligature-induced periodontitis. No data in expression of miR-10a between healthy and disease |
| Luan et al. 2018 | Literature review |
| Luo et al. 2018 | Literature review |
| Matsui et al. 2018 | Explore miRNA-200b expression in human gingival fibroblasts. No test group was reported |
| Molteni et al. 2018 | Explore miRNA expression only from periodontal pathogenes |
| Naqvi et al. 2018 | Literature review |
| Peng et al. 2018 | Explore miRNA-758 expression in PDLSC from healthy and periodontitis affected patients. Fold changes could not be calculated from Figure |
| Schulz et al. 2018 | Explore lncRNA polymorphisms in serum from patients affected with periodontitis |
| Su et al. 2018 | No experimental periodontitis was used in the animal model |
| Yang et al. 2018a | Explore miRNA-646 expression in human derived PDL cells harvested from extracted teeth. No test group was reported |
| Yang et al. 2018b | Explore miRNA-21 expression in serum-derived human monocytes and periodontal pathogens. No test group was reported |
| Acharya et al. 2019 | Systematic review |
| Akkouch et al. 2019 | Use miR-200c as a drug to mediate bone loss in a LPS-induced periodontitis (rats). Fold change was not reported between PBS (control) and untreated (LPS) group |
| Asa'ad et al. 2019 | Literature review |
| Baus-Dominguez et al. 2019 | Not exploring miRNAs |
| Chen et al. 2019 | Explore miRNA expression of miR-21, miR-103a and miR-182 from human derived PDL stem cells harvested from extracted teeth. No test group was reported |
| Du et al. 2019 | Explore miRNA expression in human derived PDL stem cells harvested from extracted teeth. No test group was reported |
| Han et al. 2019a | No experimental periodontitis was used in the animal model |
| Han et al. 2019b | Explore miRNA-132 expression in human derived PDL stem cells harvested from extracted teeth from both healthy and periodontitis patients. However, no fold changes between groups were reported prior LPS induction. |
| Lei et al. 2019 | Literature review |
| Li et al. 2019 | Re-analysis of data from three studies (Ogata et al. 2014, Stoecklin-Wasmer et al. 2012, Xie et al 2011) |
| Li et al. 2019b | Explore MALAT1 and miRNA-20a expression in human gingival fibroblasts. No test group was used for experiments evaluating miR-20a. |
| Liu et al. 2019 | Explore lncRRA expression in human PDL stem cells |
| Menini et al. 2019 | Explore miRNA expression in peri-implantitis lesions. No fold changes or details on up- or downregulation were provided. |
| Tang et al. 2019 | Explore miRNA-146a expression in human derived PDL fibroblasts harvested from extracted teeth. No test group was reported |
| Wang et al. 2019a | Explore miRNA-181b-5p role in periapical lesions, not periodontal disease. |
| Wang et al. 2019b | Not exploring miRNAs |
| Wu et al. 2019 | Explore miRNA-30a expression in human derived PDL cells harvested from extracted teeth. No test group was reported |
| Zheng et al. 2019a | Explore miRNA-22-3p expression in PDL cells. No test group was reported |

| | |
|---|---|
| Zheng et al. 2019b | Explore miRNA-155-5p expression in PDLSC harvested from extracted teeth in healthy and periodontitis-affected patients. No fold changes were reported |
| Emfietzoglou et al. 2020 | Literature review |
| Wang et al. 2020 | Explore miRNA-325-3p expression in cementoblasts. No test group was reported |

**Supplementary Table S4. Characteristics of human studies reporting miRNA expression in periodontal disease**

| Authors | Type of study | Sample size (subjects) | Groups | Age (years) | Smokers | Perio therapy | PD (mm) | CAL (mm) | BOP (%) | Method | Arrays |
|---|---|---|---|---|---|---|---|---|---|---|---|
| Amaral et al. 2019 | Cross-sectional | 9 | CP | 49.6 (8.1) | No | No | 2.3 (0.5) | 2.8 (0.6) | 33.8 (17.5) | Gingival biopsies | Microarray, RT-PCR |
| | | 9 | AP | 37.0 (5.5) | | | 3.0 (1.0) | 3.4 (0.9) | 28.8 (20.5) | | |
| Bagavad Gita et al. 2019 | Case-control | 66 | Healthy (Control) | 48.0 (4.4) | N/A | N/A | N/A | N/A | N/A | Serum | RT-PCR |
| | | 66 | ACS | 52.1 (2.5) | 32.0% | | | | | | |
| | | 66 | ACS + Moderate/Severe CP | 58.2 (1.2) | 41.6% | | | | | | |
| | | 66 | Moderate/Severe CP | 43.2 (1.9) | N/A | | | | | | |
| Bao et al. 2019 | Cross-sectional | 66 | ACS | 52.1 (2.5) | 32.0% | N/A | N/A | N/A | N/A | PDLSC from extracted teeth | RT-PCR |
| | | 66 | ACS + Moderate/Severe CP | 58.2 (1.2) | 41.6% | | | | | | |
| Chen et al. 2019 | Cross-sectional | 26 | Healthy (Control) | N/A | No | Yes (Only patients with CP) | N/A | N/A | N/A | Gingival biopsies | RT-PCR |
| | | | Chronic Periodontitis | | | | | | | | |
| Fujimori et al. 2019 | Cross-sectional | 26 | No/Mild CP | 63.3 (13.9) | 6 (5.0%) | Yes (SPT) | 1.9 (0.2) | 1.9 (0.2) | 6.9 (9.2) | Unstimulated saliva | Microarray, RT-PCR |
| | | 58 | Moderate CP | 68.6 (8.7) | | | 2.0 (0.3) | 2.6 (0.7) | 5.8 (4.9) | | |
| | | 36 | Severe CP | 71.7 (7.9) | | | 2.3 (0.5) | 3.9 (1.4) | 11.3 (12.7) | | |
| Ghotloo et al. 2019 | Cross-sectional | 10 | Healthy (Control) | 32.0 (12.0) | No | Yes (SRP) | 2.0 (0.5) | 2.1 (0.5) | N/A | Gingival biopsies | RT-PCR |
| | | 18 | AP | 27.0 (13.0) | | | 5.6 (1.6) | 7.1 (1.9) | | | |
| He et al. 2018 | Case-control | 150 | Healthy (Control) | 29.0 (12.0) | 17 (11.3%) | N/A | 1.7 (1.4) | 0.8 (1.1) | 8.9 (0.6) | Serum | RT-PCR |
| | | 120 | AP | 27.0 (10.0) | 38 (31.7%) | | 6.4 (1.7) | 6.7 (1.3) | 82.1 (8.5) | | |
| Jia et al. 2020 | Cross-sectional | 18 | Healthy (Control) | N/A | N/A | No | N/A | N/A | N/A | Gingival biopsies | qRT-PCR |
| | | 24 | CP | | | Yes | | | | | |
| Kalea et al. 2015 | Cross-sectional | 17 | Normal weight subjects with Severe Periodontitis | 50.3 (9.4) | N/A | No | N/A | N/A | N/A | Gingival biopsies | Microarray, RT-PCR |
| | | 19 | Obese subjects with Severe Periodontitis | 50.5 (4.9) | | | | | | | |

| | | | | | | | | | | | |
|---|---|---|---|---|---|---|---|---|---|---|---|
| Lee et al. 2011 | Cross-sectional | N/A | Healthy (Control) | N/A | N/A | Yes (Only patients with CP) | N/A | N/A | N/A | Gingival biopsies | Microarray, RT-PCR |
| | | | CP | | | | | | | | |
| Li et al. 2018 | Cross-sectional | N/A | Healthy (Control) | 18-50 | No | N/A | N/A | N/A | N/A | PDLSC from extracted teeth | RT-PCR |
| | | | Periodontitis | | | | | | | | |
| Liu et al. 2011 | Cross-sectional | 8 | Healthy (Control) | 29-38 | No | N/A | N/A | N/A | N/A | PDLSC from extracted teeth | RT-PCR |
| | | 7 | Chronic Periodontitis | 31-42 | | | | | | | |
| Liu et al. 2019 | Cross-sectional | 20 | Healthy (Control) | 18-25 | N/A | N/A | N/A | N/A | N/A | PDLSC from extracted teeth | RT-PCR |
| | | 20 | Chronic Periodontitis | 24-38 | | | | | | | |
| Mico-Martinez et al. 2018 | Cross-sectional | 9 | Healthy (Control) | 33.3 (12.1) | No | No | 2.0 (0.7) | 2.0 (0.7) | N/A | GCF | RT-PCR |
| | | 9 | Moderate/Severe CP | 50.4 (8.1) | | | 5.9 (0.6) | 7.0 (1.0) | | | |
| Motedayyen et al. 2015 | Cross-sectional | 10 | Healthy (Control) | 32.0 (12.0) | No | No | 2.0 (0.5) | 2.1 (0.6) | N/A | Gingival biopsies | RT-PCR |
| | | 20 | CP | 44.0 (8.0) | | Yes (SRP) | 4.6 (1.3) | 5.9 (1.4) | | | |
| Na et al. 2016 | Cross-sectional | N/A | Healthy (Control) | 41.7 (12.3) | N/A | N/A | <3 | <3 | N/A | Gingival biopsies | Microarray, RT-PCR |
| | | 14 | Periodontitis | 46.8 (5.89) | | | 7.1 (1.0) | 8.1 (0.7) | | | |
| Naqvi et al. 2019 | Cross-sectional | 8 | Normal weight subjects with Healthy Periodontium | N/A | N/A | Yes (Only patients with CP) | N/A | N/A | N/A | Gingival biopsies | Microarray, RT-PCR |
| | | 8 | Normal weight subjects with Severe Periodontitis | N/A | | | | | | | |
| | | 6 | Obese subjects with Healthy Periodontium | 35.3 (N/A) | | | | | | | |
| | | 6 | Obese subjects with Severe Periodontitis | 40.0 (N/A) | | | | | | | |
| Nisha et al. 2019 | Cross-sectional | 16 | Healthy (Control) | 40.6 (8.5) | No | No | 1.6 (0.4) | 0.4 (0.4) | 8.0 (1.8) | Saliva | Next Generation Sequencing RT-PCR |
| | | 16 | Moderate/Severe CP | 43.4 (9.9) | | | 4.8 (1.0) | 5.9 (1.0) | 95.7 (3.7) | | |
| Ogata et al. 2014 | Cross-sectional | 3 | Healthy (Control) | N/A | N/A | No | N/A | N/A | 0 | Gingival biopsies | Microarray, RT-PCR |
| | | 3 | CP | | | Yes | 7.3 (1.5 | 9.3 (1.2) | 100 | | |
| Ou et al. 2019 | Cross-sectional | 50 | Healthy (Control) | 13-76 | N/A | N/A | 2.3 (0.2) | 2.4 (0.3) | 16.2 (1.7) | Gingival biopsies and GCF | |

| | | | | | | | | | | | |
|---|---|---|---|---|---|---|---|---|---|---|---|
| | | 40 | Diabetes | | | | 2.5 (0.3) | 2.6 (0.2) | 22.9 (3.2) | | RT-PCR (Gingival samples) |
| | | 45 | Periodontitis | | | | 4.1 (0.6) | 4.0 (0.3) | 52.1 (5.5) | | |
| | | 63 | Periodontitis and Diabetes | | | | 5.6 (0.7) | 5.1 (0.3) | 67.6 (5.9) | | |
| Perri et al. 2012 | Cross-sectional | 5 | Normal weight subjects with Healthy Periodontium | 39.4 (10.0) | Past: 7 (35%) | Yes (Only patients with CP) | N/A | N/A | N/A | Gingival biopsies | Microarray, RT-PCR |
| | | 5 | Normal weight subjects with CP | 46.6 (16.7) | | | | | | | |
| | | 5 | Obese subjects with Healthy Periodontium | 49.4 (15.4) | | | | | | | |
| | | 5 | Obese subjects with CP | 48.8 (13.3) | | | | | | | |
| Pettiette et al. 2019 | Cross-sectional | 8 | Healthy (Control) | N/A | N/A | Yes (Only patients with CP) | N/A | N/A | N/A | Gingival biopsies | Microarray |
| | | 3 | CP | 51.0 (17.4) | | | | | | | |
| Radovic et al. 2018 | Prospective | 24 | Healthy Periodontium | 33.4 | No | Yes (SRP) | 2.1 (0.3) | 2.2 (0.5) | 8.7 (5.4) | GCF | RT-PCR |
| | | 24 | CP | 54.7 | | | 4.4 (0.2) | 4.9 (0.1) | 76.5 (8.7) | | |
| | | 24 | Healthy Periodontium and Diabetes | 33.2 | | | 2.4 (0.4) | 2.4 (0.4) | 9.6 (4.3) | | |
| | | 24 | CP and Diabetes | 54.9 | | | 4.8 (0.5) | 5.2 (1.3) | 72.5 (8.3) | | |
| Saito et al. 2017 | Cross-sectional | 11 | Healthy (Control) | 32.5 (8.6) | 2 (10%) | No | 1.9 (0.2) | 2.2 (0.2) | 2.6 (1.1) | GCF and Serum | Microarray, RT-PCR |
| | | 7 | CP | 67.7 (12.3) | | Yes (SRP) | 2.9 (0.7) | 6.3 (0.9) | 32.7 (31.8) | | |
| | | 2 | AP | 37.5 (5.0) | | | 3.4 (0.4) | 6.7 (1.1) | 49.7 (2.9) | | |
| Stoecklin-Wasmer et al. 2012 | Cross-sectional | 86 | Healthy (Control) | N/A | N/A | N/A | N/A | N/A | N/A | Gingival biopsies | Gene set Enrichment analysis RT-PCR |
| | | | Periodontitis | | | | | | | | |
| Venugopal et al. 2018 | Cross-sectional | 100 | Healthy (Control) | 40.4 (8.5) | No | N/A | 2.3 (0.2) | 0.0 (0.0) | N/A | Gingival biopsies | RT-PCR |
| | | 100 | CP | 48.4 (11.6) | | | 4.4 (0.7) | 4.8 (2.4) | | | |
| Xie et al. 2011 | Cross-sectional | 10 | Healthy (Control) | 22-63 | No | No | N/A | N/A | N/A | Gingival biopsies | Microarray, RT-PCR |
| | | 10 | CP | | | | | | | | |

| | | | | | | | | | | | |
|---|---|---|---|---|---|---|---|---|---|---|---|
| Yagnik et al. 2019 | Cross-sectional | 30 | Healthy (Control) | 51.1 (7.9) | N/A | N/A | 2.3 (0.3) | 2.3 (0.3) | 44.3 (8.2) | Subgingival biofilm | RT-PCR |
| | | 30 | CP | 52.3 (7.1) | | | 6.2 (0.6) | 6.5 (0.6) | 73.7 (5.7) | | |
| | | 30 | CP and Coronary Heart Disease | 53.1 (7.7) | | | 6.3 (0.7) | 6.7 (0.7) | 75.9 (6.5) | | |
| Yoneda et al. 2019 | Case-control | 30 | Healthy (Control) | 65.0 (13.2) | Yes | N/A | 1.7 (0.2) | 1.8 (0.2) | 2.2 (2.4) | Serum | Microarray, RT-PCR |
| | | 30 | Periodontitis | 67.0 (11.7) | | | 2.2 (0.3) | 2.7 (0.7) | 14.3 (13.7) | | |
| Zhang et al. 2019 | Cross-sectional | 21 | Healthy (Control) | 41.5 (2.4) | N/A | N/A | N/A | N/A | 5.3 (2.8) | GCF | RT-PCR |
| | | 29 | CP | 37.8 (2.0) | | | | | 62.2 (18.4) | | |
| Zhao et al. 2019 | Cross-sectional | 30 | Healthy (Control) | N/A | N/A | N/A | N/A | N/A | N/A | Serum | RT-PCR |
| | | 38 | Periodontitis | | | | | | | | |
| Zhou et al. 2018 | Cross-sectional | 7 | Healthy (Control) | N/A | N/A | N/A | N/A | N/A | N/A | PDLSC from extracted teeth | RT-PCR |
| | | 7 | Periodontitis | | | | | | | | |

*N/A: Not available, CP: Chronic Periodontitis, AP: Aggressive Periodontitis, ACS: Acute coronary syndrome, SPT: Supportive periodontal therapy, SRP: Scaling and root planing, PDLSC: Periodontal ligament stem cells, GCF: Gingival crevicular fluid, RT-PCR: real-time polymerase chain reaction.*

**Supplementary Table S5. Characteristics of in vivo (animal) studies reporting miRNA expression in periodontal and peri-implant disease**

*Periodontitis*

| Authors | Type of study | Sample size (animal) | Groups | Periodontitis model | Experimental phase (weeks) | Assessment | Method | miRNA markers |
|---|---|---|---|---|---|---|---|---|
| Guo et al. 2019 | Animal (Wistar rats) | N/A | Healthy (Control)<br>Periodontitis | Ligature-induced | 5.7 | Gingival biopsies | RT-PCR | miR-218 |
| Lian et al. 2020 | Animal (mice) | 26 | Alveolar bone<br>Gingiva | Ligature-induced | 2 | Block samples (Histology) | RT-PCR | miR-335-5p |
| Nahid et al. 2011 | Animal (mice) | 10<br>15 | Healthy (Control)<br>Periodontitis | Polymicrobial inocula (*P.g., T. d. and T. f.*) | 16 | Block samples (Histology) | RT-PCR | miR-132, miR-146a and miR-155 |
| Nayar et al. 2016 | Animal (Sprague-Dawley rats) | 6<br>6 | Healthy (Control)<br>Periodontitis | Polymicrobial inocula (P.g., T. d. and T. f.) | 12 | Block samples (Histology) | RT-PCR | miR-132, miR-146a and miR-155 |
| Sugiura et al. 2020 | Animal (Wistar rats) | 8<br>8 | Healthy (Control)<br>Periodontitis | Ligature-induced | 4 | Serum | Microarray | Broad spectrum |
| Sun et al. 2014 | Animal (Rhesus monkeys) | 12<br>19<br>15 | Healthy (Control)<br>Metabolic syndrome<br>Metabolic syndrome + Periodontitis | Naturally-ocurring | N/A | Gingival biopsies | RT-PCR | Broad spectrum |
| Tomofuji et al. 2016 | Animal (Wistar rats) | 16<br>16 | Healthy (Control)<br>Periodontitis | Ligature-induced | 2 and 4 | Serum | Microarray, RT-PCR | Broad spectrum |
| Xu et al. 2016 | Animal (rats) | 2<br>2<br>2<br>2 | Healthy (Control)<br>Obesity + Healthy Periodontium<br>Periodontitis<br>Obesity + Periodontitis | Ligature-induced | 8 | Serum | RT-PCR | miR-126, miR-142-3p, miR-147, miR-155, miR-203 and miR-223 |
| Zhou et al. 2016 | Animal (Wistar rats) | N/A | Healthy (Control)<br>Periodontitis | Ligature-induced and polymicrobial inocula | 2 | Block samples (Histology) | RT-PCR | miR-138 |

*Peri-implantitis*

| Authors | Type of study | Sample size (animal) | Sample size (implants) | Implant type/surface | Groups | Periodontitis model | Experimental phase (weeks) | Assessment | Method | miRNA markers |
|---|---|---|---|---|---|---|---|---|---|---|
| Wu et al. 2017 | Animal (Labrador dogs) | 6 | 24 | ITI Tissue Level | Healthy<br>Experimental peri-implantitits | Ligature-induced | 8 | Gingival tissues | Microarray, RT-PCR | Broad spectrum |

| | | | | | | | | | | |
|---|---|---|---|---|---|---|---|---|---|---|
| Wu et al. 2019 | Animal (Labrador dogs) | 5 | 20 | ITI Tissue Level | Healthy Experimental peri-implantitits | Ligature-induced | 8 | Gingival tissues | RT-PCR | Broad spectrum |

*RT-PCR: real-time polymerase chain reaction*

**Supplementary Table S6. Quality assessment of animal research based on ARRIVE guidelines and predefined grading scores (Schwarz et al., 2012)**

| **Publication** | **5** | **6** | **7** | **8** | **9** | **10** | **11** | **12** | **13** | **Risk of Bias** |
|---|---|---|---|---|---|---|---|---|---|---|
| ***Experimental Periodontitis*** | | | | | | | | | | |
| Guo et al. 2019 | 1 | 1 | 2 | 1 | 2 | 0 | 0 | 1 | 2 | Unclear |
| Lian et al. 2020 | 2 | 1 | 2 | 1 | 1 | 1 | 0 | 2 | 2 | Unclear |
| Nahid et al. 2011 | 2 | 1 | 2 | 2 | 2 | 1 | 0 | 2 | 2 | Low |
| Nayar et al. 2016 | 2 | 1 | 2 | 2 | 2 | 1 | 1 | 2 | 2 | Low |
| Sugiura et al. 2020 | 2 | 1 | 1 | 1 | 2 | 1 | 1 | 2 | 2 | Unclear |
| Sun et al. 2014 | 2 | 1 | 2 | 2 | 2 | 2 | 0 | 2 | 2 | Low |
| Tomofuji et al. 2016 | 2 | 2 | 2 | 2 | 2 | 2 | 1 | 2 | 2 | Low |
| Xu et al. 2016 | 2 | 1 | 1 | 2 | 2 | 1 | 1 | 2 | 1 | Unclear |
| Zhou et al. 2016 | 2 | 1 | 2 | 2 | 2 | 1 | 0 | 2 | 2 | Low |
| ***Experimental Peri-implantitis*** | | | | | | | | | | |
| Wu et al. 2017 | 2 | 1 | 2 | 1 | 2 | 1 | 0 | 2 | 1 | Unclear |
| Wu et al. 2019 | 2 | 1 | 2 | 1 | 1 | 1 | 1 | 2 | 2 | Unclear |

**Supplementary Table S7. Quality assessment of non-randomized studies based on the Newcastle-Ottawa Scale (NOS).**

| | Selection | | | | Comparability | Outcome | | | |
|---|---|---|---|---|---|---|---|---|---|
| ***Cohort studies*** | Representativeness of the exposed cohort | Selection of the non-exposed cohorts | Ascertainment of exposure | Demonstration that outcome of interest was not present at the start of the study | Comparability of cohorts on the bases of the design or analysis | Ascertainment of outcome | Follow-up long enough for outcomes to occur | Adequacy of follow-up of cohorts | **Total stars** |
| Radovic et al. 2018 | * | * | * | / | * | * | * | * | 7 |

| | Selection | | | | Comparability | Exposure | | | |
|---|---|---|---|---|---|---|---|---|---|
| ***Case control studies*** | Representativeness of cases | Selection of controls | Adequate case definition | Definition of controls | Comparability of cases and controls on bases of the design or analysis | Ascertainment of exposure | Same method of ascertainment for cases and controls | Non-response rate | **Total stars** |
| Bagavad Gita et al. 2019 | / | / | / | / | * | * | * | / | 3 |
| He et al. 2018 | * | * | * | * | * | * | * | / | 7 |
| Yoneda et al. 2019 | * | * | / | / | / | * | * | / | 4 |

*<u>Note:</u> A study can be awarded a maximum of one star for each numbered item within the Selection and Outcome categories. A maximum of two stars can be given for Comparability. /: Study did not meet criteria, *: Study met criteria.*

**Supplementary Table S8. Quality assessment of human case reports, case series and cross-sectional studies based on The Joanna Briggs Institute (JBI) critical appraisal checklist.**

*Cross sectional*

| **Publication** | **1** | **2** | **3** | **4** | **5** | **6** | **7** | **8** | **Risk of Bias** |
|---|---|---|---|---|---|---|---|---|---|
| Amaral et al. 2019 | 1 | 1 | 0 | 1 | 1 | 1 | 2 | 2 | Unclear |
| Bao et al. 2019 | 1 | 1 | 0 | 0 | 0 | 0 | 2 | 1 | High |
| Chen et al. 2019 | 2 | 1 | 1 | 1 | 1 | 0 | 2 | 1 | Unclear |
| Fujimori et al. 2019 | 1 | 1 | 0 | 1 | 2 | 2 | 2 | 2 | Low |
| Ghotloo et al. 2019 | 2 | 1 | 1 | 2 | 2 | 2 | 1 | 2 | Low |
| Jia et al. 2020 | 0 | 0 | 1 | 1 | 0 | 1 | 2 | 1 | High |
| Kalea et al. 2015 | 2 | 2 | 1 | 1 | 1 | 1 | 2 | 2 | Low |
| Lee et al. 2011 | 1 | 1 | 0 | 0 | 1 | 0 | 2 | 1 | Unclear |
| Li et al. 2018a | 0 | 0 | 0 | 0 | 1 | 0 | 2 | 1 | High |
| Liu et al. 2011 | 1 | 0 | 1 | 1 | 0 | 0 | 2 | 1 | Unclear |
| Liu et al. 2019 | 0 | 1 | 0 | 0 | 0 | 0 | 2 | 1 | High |
| Mico-Martinez et al. 2018 | 2 | 2 | 1 | 2 | 2 | 2 | 2 | 1 | Low |
| Motedayyen et al. 2015 | 2 | 2 | 1 | 2 | 2 | 2 | 2 | 2 | Low |
| Na et al. 2016 | 2 | 1 | 1 | 1 | 1 | 1 | 2 | 1 | Unclear |
| Naqvi et al. 2019 | 2 | 1 | 1 | 1 | 1 | 1 | 2 | 1 | Unclear |
| Nisha et al. 2019 | 2 | 2 | 1 | 2 | 2 | 2 | 2 | 2 | Low |
| Ogata et al. 2014 | 2 | 1 | 1 | 1 | 2 | 2 | 2 | 1 | Unclear |
| Ou et al. 2019 | 1 | 1 | 1 | 1 | 0 | 0 | 2 | 1 | Unclear |
| Perri et al. 2012 | 2 | 1 | 0 | 1 | 2 | 1 | 2 | 1 | Unclear |
| Pettiette et al. 2019 | 1 | 1 | 1 | 0 | 1 | 1 | 1 | 2 | Unclear |
| Saito et al. 2017 | 2 | 2 | 1 | 1 | 2 | 2 | 2 | 2 | Low |
| Stoecklin-Wasmer et al. 2012 | 2 | 0 | 1 | 1 | 0 | 0 | 2 | 2 | Unclear |
| Venugopal et al. 2018 | 2 | 2 | 1 | 1 | 2 | 2 | 2 | 2 | Low |
| Xie et al. 2011 | 2 | 0 | 0 | 0 | 1 | 1 | 2 | 1 | Unclear |
| Yagnik et al. 2019 | 2 | 1 | 1 | 1 | 0 | 0 | 1 | 2 | Unclear |
| Zhang et al. 2019 | 2 | 1 | 1 | 0 | 0 | 0 | 1 | 1 | Unclear |
| Zhao et al. 2019 | 1 | 0 | 0 | 0 | 0 | 0 | 2 | 1 | High |
| Zhou et al. 2018 | 0 | 1 | 0 | 0 | 0 | 0 | 2 | 1 | High |

**Supplementary Table S9. Overall miRNA fold expression changes from human studies based on microarray and RT-PCR reported values.**

| miRNA | Fold change difference | | miRNA Expression | miRNA | Fold change difference | | miRNA Expression | miRNA | Fold change difference | | miRNA Expression | miRNA | Fold change difference | | miRNA Expression |
|---|---|---|---|---|---|---|---|---|---|---|---|---|---|---|---|
| | *Microarray* | *RT-PCR* | | | *Microarray* | *RT-PCR* | | | *Microarray* | *RT-PCR* | | | *Microarray* | *RT-PCR* | |
| **let-7a** | 6.5 | 4.0 | Up | **miR-107** | -2.5 | N/A | Down | **miR-302a** | 9.5, -3.5 | N/A | Up/Down | **miR-650** | 1.7 | N/A | Up |
| **let-7a-5p** | 2.2, -1.6 | N/A | Up/Down | **miR-122** | 3.5 | N/A | Up | **miR-302b** | 6.5 | N/A | Up | **miR-652** | 1.2 | N/A | Up |
| **let-7b** | 5.9 | N/A | Up | **miR-125a-3p** | 1.5 | N/A | Up | **miR-302c** | 9.5, -3.5 | N/A | Up/Down | **miR-654-3p** | -1.3 | N/A | Down |
| **let-7b-5p** | 2.5, -2.4 | N/A | Up/Down | **miR-125a-5p** | 4.7, N/A | N/A | Up/Down | **miR-302d** | 3.5 | N/A | Up | **miR-654-5p** | 1.1 | N/A | Up |
| **let-7c** | 9.5, -0.7 | N/A | Up/Down | **miR-125b** | 4.8, -0.6 | N/A | Up/Down | **miR-320a** | N/A | N/A | Down | **miR-656** | 9.5, -1.1 | N/A | Up/Down |
| **let-7c-5p** | 3.1, -1.1 | N/A | Up/Down | **miR-125b-2** | 0.8, -0.4 | N/A | Up/Down | **miR-323a-3p** | 1.2 | 2 | Up | **miR-659** | 1.1 | N/A | Up |
| **let-7d** | 4.7 | N/A | Up | **miR-125b-5p** | N/A | N/A | Down | **miR-323-5p** | -3.5 | N/A | Down | **miR-663** | 1.6 | N/A | Up |
| **let-7d-5p** | 1.7 | N/A | Up | **miR-126** | 3.8 | 6.3 | Up | **miR-335** | 3.5 | N/A | Up | **miR-664a-3p** | 1.5 | N/A | Up |
| **let-7e** | 4.7 | N/A | Up | **miR-126-3p** | 2.6 | N/A | Up | **miR-338-3p** | 3.5 | N/A | Up | **miR-671-5p** | 2.0 | N/A | Up |
| **let-7e-5p** | 2.1, N/A | N/A | Up/Down | **miR-126-5p** | N/A | N/A | Up | **miR-340** | 4.4 | N/A | Up | **miR-720** | 1.0 | N/A | Up |
| **let-7f** | 3.7 | 1.8 | Up | **miR-127-3p** | 1.3, -0.5 | N/A | Up/Down | **miR-342-3p** | 1.3 | N/A | Up | **miR-760** | 1.0 | N/A | Up |
| **let-7f-5p** | 5.5, -3.5 | N/A | Up/Down | **miR-128** | 4.2 | N/A | Up | **miR-345** | 1.1 | N/A | Up | **miR-764** | -1.3 | N/A | Down |
| **let-7g** | 3.0 | N/A | Up | **miR-129** | 0.9 | N/A | Up | **miR-361-3p** | 1.2 | N/A | Up | **miR-765** | 1.4, -1.2 | N/A | Up/Down |
| **let-7g-5p** | 4.0 | N/A | Up | **miR-129-3p** | 0.9 | N/A | Up | **miR-362-3p** | 7.5 | N/A | Up | **miR-766** | 1.7 | N/A | Up |
| **let-7i** | 3.1 | N/A | Up | **miR-130a** | 7.5 | 2.2 | Up | **miR-363** | 2.6 | N/A | Up | **miR-769-3p** | 3.5 | N/A | Up |
| **let-7i-5p** | 1.9, -3.2 | N/A | Up/Down | **miR-130a-5p** | 1.7 | N/A | Up | **miR-369-5p** | 3.5 | N/A | Up | **miR-875-3p** | 9.5 | N/A | Up |
| **miR-7-2-3p** | -1.3 | N/A | Down | **miR-130b** | 3.1 | N/A | Up | **miR-371-5p** | 1.8 | N/A | Up | **miR-886-3p** | -0.9 | N/A | Down |
| **miR-7-5p** | 5.0 | N/A | Up | **miR-132** | 2.4, -1.4 | N/A | Up/Down | **miR-372** | 9.5, -1.2 | N/A | Up/Down | **miR-886-5p** | 3.5 | N/A | Up |
| **miR-9** | 3.0 | N/A | Up | **miR-135b** | 1.9 | N/A | Up | **miR-373** | 9.5 | N/A | Up | **miR-889** | 3.5 | N/A | Up |
| **miR-10a** | 1.1 | N/A | Up | **miR-136** | 2.1 | N/A | Up | **miR-374a** | 3.0 | N/A | Up | **miR-892b** | 0.9 | N/A | Up |
| **miR-10a-5p** | 3.8 | N/A | Up | **miR-138-2** | -3.5 | N/A | Down | **miR-374a-5p** | 3.6 | N/A | Up | **miR-922** | 3.5 | N/A | Up |
| **miR-10b** | 3.5 | N/A | Up | **miR-139-3p** | 1.4 | N/A | Up | **miR-374b** | 3.0 | N/A | Up | **miR-933** | 0.9 | N/A | Up |
| **miR-10b-5p** | 2.2 | N/A | Up | **miR-139-5p** | 1.1 | N/A | Up | **miR-375** | 0.8 | N/A | Up | **miR-936** | 1.1 | N/A | Up |
| **miR-15a** | 1.6 | 1 | Up | **miR-140-3p** | 1.9 | N/A | Up | **miR-376a** | -0.5 | N/A | Down | **miR-939** | 1.3 | N/A | Up |

| | | | | | | | | | | | | | | | |
|---|---|---|---|---|---|---|---|---|---|---|---|---|---|---|---|
| **miR-15a-5p** | 3.2, -0.7 | N/A | Up/Down | **miR-140-5p** | 3.5 | N/A | Up | **miR-376c** | -0.5 | N/A | Down | **miR-1181** | -3.5 | N/A | Down |
| **miR-15b** | 2.0, -0.8 | N/A | Up/Down | **miR-141** | 2.0 | N/A | Up | **miR-378** | 0.8 | N/A | Up | **miR-1183** | -1.4 | N/A | Down |
| **miR-16** | 2.0 | N/A | Up | **miR-141-3p** | 2.8, -3.2 | N/A | Up/Down | **miR-378a-3p** | 4.5 | N/A | Up | **miR-1202** | 1.5 | N/A | Up |
| **miR-16-5p** | 3.9, -1.1 | N/A | Up/Down | **miR-142-3p** | 3.2 | 3.3 | Up | **miR-378c** | 4.1 | N/A | Up | **miR-1205** | -3.5 | N/A | Down |
| **miR-17** | 1.8 | 2.7, -0.3 | Up/Down | **miR-143** | 1.2 | N/A | Up | **miR-379** | 3.5, -0.4 | N/A | Up/Down | **miR-1207-5p** | 3.5 | N/A | Up |
| **miR-17-3p** | 2.5 | N/A | Up | **miR-143-3p** | 4.6 | 0.4 | Up | **miR-381** | 6.7, -0.5 | N/A | Up/Down | **miR-1224-5p** | 0.6 | N/A | Up |
| **miR-17-5p** | 2.0 | N/A | Up | **miR-144** | 4.1 | 5.3 | Up | **miR-381-3p** | 3.6 | 0.4 | Up | **miR-1225-3p** | 3.5 | N/A | Up |
| **miR-18a** | 1.3 | 0.1 | Up | **miR-144-3p** | 2.2 | -0.3 | Up/Down | **miR-409-3p** | -1.5 | N/A | Down | **miR-1226-5p** | N/A | -3.5 | Down |
| **miR-18b** | 3.5 | N/A | Up | **miR-144-5p** | N/A | N/A | Up | **miR-410** | 5.3 | N/A | Up | **miR-1229** | 0.9 | N/A | Up |
| **miR-19a** | 2.6 | 2.1 | Up | **miR-145** | 1.3 | N/A | Up | **miR-411** | 3.5 | N/A | Up | **miR-1238** | -1.3 | N/A | Down |
| **miR-19a-3p** | 7.9 | N/A | Up | **miR-145-5p** | N/A | N/A | Up | **miR-423-3p** | 3.4 | N/A | Up | **miR-1246** | 0.3 | N/A | Up |
| **miR-19b** | 4.8 | 3.8 | Up | **miR-146a** | 1.6 | 10.4, -1.6 | Up/Down | **miR-423-5p** | 2.1 | N/A | Up | **miR-1253** | 1.2 | N/A | Up |
| **miR-19b-3p** | 2.2 | N/A | Up | **miR-146a-5p** | 4.0 | N/A | Up | **miR-424** | 2.0 | N/A | Up | **miR-1257** | 3.5 | N/A | Up |
| **miR-20a** | 4.1 | 3.8 | Up | **miR-146b** | 1.8 | 2.2 | Up | **miR-424-5p** | 5.5 | N/A | Up | **miR-1259** | 3.5 | N/A | Up |
| **miR-20a-3p** | N/A | N/A | Up | **miR-148a** | 1.7 | 0.8 | Up | **miR-425** | 1.3 | N/A | Up | **miR-1260** | 0.4 | N/A | Up |
| **miR-20a-5p** | 4.5 | N/A | Up | **miR-148b** | 1.4 | N/A | Up | **miR-429** | 2.7 | N/A | Up | **miR-1260b** | 0.5 | N/A | Up |
| **miR-20b** | 2.4 | N/A | Up | **miR-149** | 0.8 | N/A | Up | **miR-432** | 1.1 | N/A | Up | **miR-1266** | 3.5 | N/A | Up |
| **miR-21** | 1.8 | 8 | Up | **miR-150** | 2.4 | 5.8 | Up | **miR-431** | -3.5 | N/A | Down | **miR-1273e** | 1.3 | N/A | Up |
| **miR-21-3p** | 2.1, N/A | N/A | Up/Down | **miR-150-5p** | 3.0 | N/A | Up | **miR-433** | -1.2 | N/A | Down | **miR-1274a** | 0.6 | N/A | Up |
| **miR-21-5p** | 7.0 | N/A | Up | **miR-151a-3p** | 3.6 | N/A | Up | **miR-449a** | 2.4 | N/A | Up | **miR-1274b** | 0.6, -3.5 | N/A | Up/Down |
| **miR-22** | 1.9 | 0.3 | Up | **miR-151a-5p** | 0.4 | N/A | Up | **miR-449b** | 9.5 | N/A | Up | **miR-1275** | -3.5 | N/A | Down |
| **miR-22-3p** | 2.4 | N/A | Up | **miR-154** | -0.5 | N/A | Down | **miR-450b-5p** | 3.5 | N/A | Up | **miR-1277** | -3.5 | N/A | Down |
| **miR-23a** | 4.8 | 3.6 | Up | **miR-155** | 1.7, -1.7 | 3.5, -1.9 | Up/Down | **miR-451** | 2.6, -3.5 | N/A | Up/Down | **miR-1280** | 0.8 | N/A | Up |
| **miR-23a-3p** | 0.1, -1.9 | N/A | Up/Down | **miR-155-5p** | 2.1, N/A | N/A | Up/Down | **miR-452** | 2.2 | N/A | Up | **miR-1284** | -1.3 | N/A | Down |
| **miR-23b** | 2.9 | N/A | Up | **miR-181a** | 1.2, -0.5 | N/A | Up/Down | **miR-454** | 2.4 | N/A | Up | **miR-1288** | 0.9 | N/A | Up |
| **miR-23b-3p** | 0.3, -1.7 | N/A | Up/Down | **miR-181b** | 2.9 | 4.0 | Up | **miR-455-3p** | 3.5 | N/A | Up | **miR-1290** | 0.9 | N/A | Up |
| **miR-24-1** | 0.8 | N/A | Up | **miR-181b-5p** | 2.0, N/A | N/A | Up/Down | **miR-483-5p** | 1.8 | N/A | Up | **miR-1297** | 3.5 | N/A | Up |

| | | | | | | | | | | | | | | | |
|---|---|---|---|---|---|---|---|---|---|---|---|---|---|---|---|
| **miR-24-3p** | 1.3 | N/A | Up | **miR-181c** | 2.4 | N/A | Up | **miR-484** | 1.2 | N/A | Up | **miR-1306-3p** | N/A | -0.4 | Down |
| **miR-25-3p** | 0.4 | N/A | Up | **miR-181d** | 1.7 | N/A | Up | **miR-485-5p** | -3.5 | N/A | Down | **miR-1324** | 9.5 | N/A | Up |
| **miR-26a** | -0.5 | N/A | Down | **miR-182** | -3.5 | N/A | Down | **miR-486-5p** | 2.5 | N/A | Up | **miR-1539** | 0.9 | N/A | Up |
| **miR-26a-5p** | 1.6 | N/A | Up | **miR-185** | 2.1 | 2.3 | Up | **miR-487a** | -3.5 | N/A | Down | **miR-1911-3p** | 1.3 | N/A | Up |
| **miR-26b** | 3.5 | N/A | Up | **miR-185-5p** | 0.3 | N/A | Up | **miR-488** | 3.5, -3.5 | N/A | Up/Down | **miR-1914** | 0.6 | N/A | Up |
| **miR-26b-5p** | 3.1 | N/A | Up | **miR-186** | 1.3 | N/A | Up | **miR-497** | 2.0 | N/A | Up | **miR-1972** | 1.1 | N/A | Up |
| **miR-27a** | 3.5 | N/A | Up | **miR-187** | -3.5 | N/A | Down | **miR-501-5p** | 1.6 | N/A | Up | **miR-2276** | -1.2 | N/A | Down |
| **miR-27a-3p** | 3.6, -1.0 | 1.8 | Up/Down | **miR-187-3p** | N/A | N/A | Down | **miR-502-5p** | -3.5 | N/A | Down | **miR-2278** | 1.1 | N/A | Up |
| **miR-27a-5p** | N/A | -0.2 | Down | **miR-188-3p** | -3.5 | N/A | Down | **miR-505** | 1.2 | N/A | Up | **miR-3128** | 1.3 | N/A | Up |
| **miR-27b** | 2.2 | N/A | Up | **miR-188-5p** | 1.4 | 0.5 | Up | **miR-507** | 3.5 | N/A | Up | **miR-3136-5p** | 1.1 | N/A | Up |
| **miR-27b-3p** | 2.0, -0.9 | N/A | Up/Down | **miR-190** | 7.5 | N/A | Up | **miR-511** | 4.3 | N/A | Up | **miR-3141** | 1.4 | N/A | Up |
| **miR-28-5p** | 1.3 | N/A | Up | **miR-192-5p** | 1.2, -1.8 | N/A | Up/Down | **miR-513b** | 5.2 | N/A | Up | **miR-3147** | 1.1 | N/A | Up |
| **miR-29a** | 2.0 | N/A | Up | **miR-193a-3p** | 0.7, -0.5 | N/A | Up/Down | **miR-513c** | 1.4, -3.5 | N/A | Up/Down | **miR-3154** | 1.1 | N/A | Up |
| **miR-29a-3p** | 3.1, -1.4 | N/A | Up/Down | **miR-193a-5p** | 0.9 | N/A | Up | **miR-514** | 3.5 | N/A | Up | **miR-3156-5p** | 0.5 | N/A | Up |
| **miR-29b** | 2.4 | N/A | Up | **miR-194** | 3.5 | N/A | Up | **miR-514b-5p** | 1.2 | N/A | Up | **miR-3180** | 1.1 | N/A | Up |
| **miR-29b-2** | 3.5 | N/A | Up | **miR-194-5p** | N/A | N/A | Up | **miR-518c** | 3.5 | N/A | Up | **miR-3195** | 0.7 | N/A | Up |
| **miR-29b-3p** | 6.7 | N/A | Up | **miR-195** | 2.5 | N/A | Up | **miR-519** | 9.5 | N/A | Up | **miR-3198** | -1.3 | N/A | Down |
| **miR-29c** | 1.9 | N/A | Up | **miR-195-5p** | 3.6 | N/A | Up | **miR-519c-3p** | 9.5 | N/A | Up | **miR-3202** | 1.1 | N/A | Up |
| **miR-29c-3p** | 8.5, -3.1 | N/A | Up/Down | **miR-196a** | -1.3 | -0.5 | Down | **miR-519d** | 9.5 | N/A | Up | **miR-3622a-5p** | -1.4 | N/A | Down |
| **miR-30a** | 3.0, -0.4 | N/A | Up/Down | **miR-196b-5p** | 1.7 | N/A | Up | **miR-520a-5p** | 3.5 | N/A | Up | **miR-3652** | 1.6 | N/A | Up |
| **miR-30a-5p** | N/A | 4.2 | Up | **miR-199a-3p** | -1.5 | N/A | Down | **miR-520b** | 3.5 | N/A | Up | **miR-3654** | 0.8 | N/A | Up |
| **miR-30b** | 3.0 | N/A | Up | **miR-199a-5p** | -0.4 | N/A | Down | **miR-520d-3p** | 8.8 | N/A | Up | **miR-3667-5p** | 1.2 | N/A | Up |
| **miR-30b-5p** | 1.3, -0.5 | 0.2 | Up/Down | **miR-199b-3p** | -1.5 | N/A | Down | **miR-520d-5p** | 9.5 | N/A | Up | **miR-3675-3p** | 0.9 | N/A | Up |
| **miR-30c** | 2.4 | N/A | Up | **miR-200a** | 1.6, -3.5 | N/A | Up/Down | **miR-520e** | 9.5 | N/A | Up | **miR-3679-5p** | 1.6 | N/A | Up |
| **miR-30c-1** | -3.5 | N/A | Down | **miR-200a-5p** | N/A | N/A | Down | **miR-523** | 3.5 | N/A | Up | **miR-3692** | 0.7 | N/A | Up |
| **miR-30c-5p** | 1.7 | N/A | Up | **miR-200b** | 1.9 | 2.7 | Up | **miR-524-5p** | 6.5 | N/A | Up | **miR-3907** | 0.9 | N/A | Up |
| **miR-30d** | 2.8 | 1.0 | Up | **miR-200b-3p** | N/A | N/A | Down | **miR-532-3p** | 3.5 | N/A | Up | **miR-3911** | 0.7 | N/A | Up |
| **miR-30e** | 2.8 | 3.2 | Up | **miR-200b-5p** | 1.3, N/A | 1.7 | Up/Down | **miR-542-5p** | -3.5 | N/A | Down | **miR-3917** | 2.1 | N/A | Up |

| | | | | | | | | | | | | | | | |
|---|---|---|---|---|---|---|---|---|---|---|---|---|---|---|---|
| **miR-30e-3p** | N/A | N/A | Up | **miR-200c** | 0.7 | N/A | Up | **miR-543** | 6.1, -3.5 | 0.4 | Up/Down | **miR-3923** | 1.2 | N/A | Up |
| **miR-30e-5p** | N/A | N/A | Up | **miR-200c-3p** | N/A | N/A | Down | **miR-545** | 9.5 | N/A | Up | **miR-3926** | 1.1 | N/A | Up |
| **miR-31** | 2.1 | N/A | Up | **miR-200c-5p** | 1.3, N/A | N/A | Up/Down | **miR-548a-3p** | 8.7 | N/A | Up | **miR-4121** | -1.6 | N/A | Down |
| **miR-31-3p** | N/A | N/A | Down | **miR-202** | 1.9 | N/A | Up | **miR-548c-3p** | 9.5 | N/A | Up | **miR-4259** | 1.2 | N/A | Up |
| **miR-32** | 7.5 | N/A | Up | **miR-203** | 1.5 | 2.2 | Up | **miR-548d-3p** | 4.3 | N/A | Up | **miR-4269** | -1.3 | N/A | Down |
| **miR-32-5p** | 2.8 | N/A | Up | **miR-203a** | N/A | N/A | Down | **miR-548e** | 9.5, -3.5 | N/A | Up/Down | **miR-4271** | 1.4 | N/A | Up |
| **miR-33a** | 2.1 | N/A | Up | **miR-204** | 0.7, -3.5 | N/A | Up/Down | **miR-551b** | 1.2 | N/A | Up | **miR-4286** | 0.6 | N/A | Up |
| **miR-33b** | -3.5 | N/A | Down | **miR-205** | 0.6, -1.8 | -2.3 | Up/Down | **miR-555** | 1.9 | N/A | Up | **miR-4291** | 0.8 | N/A | Up |
| **miR-34a** | 3.7, -0.5 | N/A | Up/Down | **miR-205-5p** | N/A | N/A | Down | **miR-557** | 1.2, -1.5 | 4 | Up/Down | **miR-4299** | 1.7 | N/A | Up |
| **miR-34a-5p** | -4.0 | N/A | Down | **miR-208b** | -3.5 | N/A | Down | **miR-563** | -1.3 | N/A | Down | **miR-4306** | 1.8 | N/A | Up |
| **miR-34c-5p** | 3.9 | N/A | Up | **miR-210** | 1.0 | 1.2, -0.4 | Up/Down | **miR-564** | 1.2 | N/A | Up | **miR-4310** | 0.9 | N/A | Up |
| **miR-92a-2** | -3.5 | N/A | Down | **miR-211** | 2.6, -0.9 | N/A | Up | **miR-575** | 1.8 | N/A | Up | **miR-4314** | 1.1 | N/A | Up |
| **miR-92a-3p** | 0.3, -0.8 | N/A | Up/Down | **miR-212** | -1.3 | N/A | Down | **miR-582-5p** | 3.5 | N/A | Up | **miR-4317** | 0.9 | N/A | Up |
| **miR-92b** | 0.9 | N/A | Up | **miR-214** | -2.0 | N/A | Down | **miR-584** | 1.1 | N/A | Up | **miR-4327** | -1.5 | N/A | Down |
| **miR-93** | 2.4 | N/A | Up | **miR-214-3p** | N/A | 2.7 | Up | **miR-587** | 3.5 | N/A | Up | **miR-4454** | 1.4 | N/A | Up |
| **miR-93-5p** | 3.1 | N/A | Up | **miR-218** | 3.5, -0.6 | N/A | Up/Down | **miR-589** | -3.5 | N/A | Down | **miR-4642** | 1.3 | N/A | Up |
| **miR-95** | 3.0 | N/A | Up | **miR-219-5p** | 2.2 | N/A | Up | **miR-590-5p** | 3.0 | N/A | Up | **miR-4683** | -1.2 | N/A | Down |
| **miR-96** | 2.1 | N/A | Up | **miR-221** | 2.2 | N/A | Up | **miR-592** | 3.5 | N/A | Up | **miR-4704-5p** | 1.3 | N/A | Up |
| **miR-98** | 2.4 | N/A | Up | **miR-221-3p** | 1.0, -3.2 | N/A | Up/Down | **miR-593** | 3.5 | N/A | Up | **miR-4717-5p** | 0.6 | N/A | Up |
| **miR-99a** | 0.6, -0.6 | N/A | Up/Down | **miR-222** | 0.8, -0.4 | -2.3 | Up/Down | **miR-597** | -3.5 | N/A | Down | **miR-4721** | -1.6 | 12 | Up/Down |
| **miR-99a-5p** | N/A | N/A | Down | **miR-222-3p** | -0.4 | N/A | Down | **miR-605** | -3.5 | N/A | Down | **miR-4725-5p** | 1.2 | N/A | Up |
| **miR-99b** | 1.2 | N/A | Up | **miR-223** | 2.4 | 4.9 | Up | **miR-607** | 9.5 | N/A | Up | **miR-6770-5p** | 0.7 | N/A | Up |
| **miR-100** | -0.6 | -1.6 | Down | **miR-223-3p** | N/A, -1.4 | N/A | Up/Down | **miR-609** | 3.5 | N/A | Up | **miR-7704** | 3.8 | N/A | Up |
| **miR-100-5p** | N/A | N/A | Down | **miR-223-5p** | N/A | -0.1 | Down | **miR-617** | 0.8 | N/A | Up | | | | |
| **miR-101** | 4.1 | N/A | Up | **miR-224** | 0.8 | N/A | Up | **miR-623** | 1.2 | N/A | Up | | | | |
| **miR-101-3p** | 4.6 | N/A | Up | **miR-299-3p** | -3.5 | N/A | Down | **miR-625** | 1.8 | N/A | Up | | | | |
| **miR-103** | 0.7 | 0.3 | Up | **miR-299-5p** | -1.3 | N/A | Down | **miR-634** | 0.9 | N/A | Up | | | | |
| **miR-103a-3p** | -2.5 | N/A | Down | **miR-300** | 9.5 | N/A | Up | **miR-642b** | 0.8, -1.4 | N/A | Up/Down | | | | |

| | | | | | | | | | | | |
|---|---|---|---|---|---|---|---|---|---|---|---|
| **miR-105** | 3.5 | 2.2 | Up | **miR-301a** | 6.1 | 3.8 | Up | **miR-644** | 3.5 | N/A | Up |
| **miR-106a** | 3.0 | N/A | Up | **miR-301a-3p** | N/A | N/A | Up | **miR-645** | 1.1 | N/A | Up |
| **miR-106b** | 4.5 | 3.8 | Up | **miR-301b** | 9.5 | N/A | Up | **miR-648** | 3.5 | N/A | Up |

**Supplementary Table S10. Overall miRNA fold expression changes from in vivo studies based on microarray and RT-PCR reported values.**

| Periodontitis (animal studies) | | | | | | | | Peri-implantitis (animal studies) | | | |
|---|---|---|---|---|---|---|---|---|---|---|---|
| **miRNA** | **Fold change difference** | | **miRNA Expression** | **miRNA** | **Fold change difference** | | **miRNA Expression** | **miRNA** | **Fold change difference** | | **miRNA Expression** |
| | *Microarray* | *RT-PCR* | | | *Microarray* | *RT-PCR* | | | *Microarray* | *RT-PCR* | |
| **let-7a-1-3p** | -0.5 | N/A | Down | **miR-217-5p** | -0.3 | N/A | Down | **let-7c** | -2.1 | N/A | Down |
| **let-7c-2-3p** | -0.5 | N/A | Down | **miR-218** | N/A | -0.8 | Down | **let-7e** | 1.7 | N/A | Up |
| **let-7d-3p** | -0.5 | N/A | Down | **miR-223** | N/A | 0.7 | Up | **let-7g** | -1.5 | -1^ | Down |
| **let-7e** | N/A | -0.4^ | Down | **miR-224-5p** | -0.6 | N/A | Down | **miR-7** | 1.7 | N/A | Up |
| **let-7g-3p** | -0.3 | N/A | Down | **miR-301a-5p** | -0.5 | N/A | Down | **miR-9** | -4.2 | N/A | Down |
| **miR-20b-3p** | -0.5 | N/A | Down | **miR-323-3p** | -0.5 | N/A | Down | **miR-16** | -1.6 | N/A | Down |
| **miR-21-3p** | 2.0 | N/A | Up | **miR-335** | -0.5 | N/A | Down | **miR-20a** | -7.5 | N/A | Down |
| **miR-23b-5p** | -0.5 | N/A | Down | **miR-335-5p** | N/A | -1 - -0.5 | Down | **miR-23a** | -2.3 | N/A | Down |
| **miR-24-1-5p** | -0.6 | N/A | Down | **miR-338** | N/A | 0.4 | Up | **miR-26a** | -1.8 | N/A | Down |
| **miR-26a-3p** | 1.6 | N/A | Up | **miR-338-5p** | 2.8 | N/A | Up | **miR-27a** | -2 | -0.4^ | Down |
| **miR-28-3p** | -0.7 | N/A | Down | **miR-344b-1-3p** | 2.0 | N/A | Up | **miR-27b** | -1.7 | N/A | Down |
| **miR-29-5p** | 1.5 | N/A | Up | **miR-344g** | -0.4 | N/A | Down | **miR-29a** | -1.8 | -0.25^ | Down |
| **miR-32-3p** | -0.5 | N/A | Down | **miR-344i** | -0.4 | N/A | Down | **miR-92b** | -2.4 | N/A | Down |
| **miR-34a-3p** | -0.6 | N/A | Down | **miR-362-3p** | -0.6 | N/A | Down | **miR-93** | -2 | N/A | Down |
| **miR-93-3p** | -0.3 | N/A | Down | **miR-374-5p** | -0.4 | N/A | Down | **miR-98** | 2.4 | N/A | Up |
| **miR-96-3p** | -0.3 | N/A | Down | **miR-376b-3p** | -0.3 | N/A | Down | **miR-101** | -1.6 | N/A | Down |
| **miR-98-3p** | -0.7 | N/A | Down | **miR-379** | N/A | 0.3^ | Up | **miR-125a** | -1.9 | N/A | Down |
| **miR-98-5p** | -0.4 | N/A | Down | **miR-421-5p** | -0.6 | N/A | Down | **miR-127** | -1.5 | N/A | Down |
| **miR-126** | N/A | 1.5^ | Up | **miR-434-5p** | -0.6 | N/A | Down | **miR-140** | -1.7 | N/A | Down |
| **miR-132** | N/A | -0.1 - 1^ | Up/Down | **miR-448-5p** | -0.3 | N/A | Down | **miR-142** | 1.7 | -0.5^ | Up |
| **miR-133a-3p** | 3.7 | N/A | Up | **miR-485** | N/A | 1.1 | Up | **miR-145** | 2 | 1.15^ | Up |
| **miR-133b-3p** | 2.6 | N/A | Up | **miR-490-3p** | -0.6 | N/A | Down | **miR-146a** | -1.7 | N/A | Down |

| | | | | | | | | | | | |
|---|---|---|---|---|---|---|---|---|---|---|---|
| **miR-137-5p** | -0.3 | N/A | Down | **miR-493-3p** | 2.5 | N/A | Up | **miR-152** | -1.5 | N/A | Down |
| **miR-138** | 1.5 | N/A | Up | **miR-493-5p** | -0.5 | N/A | Down | **miR-200a** | -1.6 | N/A | Down |
| **miR-142-3p** | 1.6 | 2.0^ | Up | **miR-495** | -0.4 | N/A | Down | **miR-204** | -1.8 | N/A | Down |
| **miR-142-5p** | 1.5 | N/A | Up | **miR-543-3p** | -0.4 | N/A | Down | **miR-211** | -2.3 | N/A | Down |
| **miR-146a** | N/A | 0.5-2 | Up | **miR-578** | N/A | -0.1 | Down | **miR-340** | -3.0 | N/A | Down |
| **miR-146b-3p** | -0.5 | N/A | Down | **miR-583** | N/A | -0.3 | Down | **miR-342** | -2.6 | N/A | Down |
| **miR-147** | N/A | 2.6^ | Up | **miR-598-3p** | -0.6 | N/A | Down | **miR-361** | -2.0 | N/A | Down |
| **miR-148a-5p** | -0.6 | N/A | Down | **miR-628** | -0.4 | N/A | Down | **miR-374a** | -3.1 | N/A | Down |
| **miR-148b-3p** | -0.4 | N/A | Down | **miR-674-5p** | 10.5 | N/A | Up | **miR-375** | 2.4 | N/A | Up |
| **miR-151-5p** | -0.4 | N/A | Down | **miR-741-3p** | -0.6 | N/A | Down | **miR-429** | -5.6 | N/A | Down |
| **miR-155** | N/A | 0.5-3^ | Up | **miR-758** | -0.5 | N/A | Down | **miR-451** | -1.9 | N/A | Down |
| **miR-181a-2-3p** | -0.4 | N/A | Down | **miR-761** | -0.5 | N/A | Down | **miR-452** | 2.5 | N/A | Up |
| **miR-183-3p** | -0.7 | N/A | Down | **miR-872-3p** | -0.4 | N/A | Down | **miR-486** | -1.5 | N/A | Down |
| **miR-187-3p** | -0.5 | N/A | Down | **miR-875** | -0.4 | N/A | Down | **miR-500** | 1.7 | N/A | Up |
| **miR-192-3p** | -0.6 | N/A | Down | **miR-1199-3p** | -0.5 | N/A | Down | **miR-532** | -2.1 | N/A | Down |
| **miR-193a-3p** | -0.5 | N/A | Down | **miR-1839-5p** | -0.6 | N/A | Down | **miR-1271** | -1.6 | N/A | Down |
| **miR-194-5p** | -0.6 | N/A | Down | **miR-3084c-3p** | 1.6 | N/A | Up | | | | |
| **miR-200b-3p** | -0.6 | N/A | Down | **miR-3570-5p** | -0.6 | N/A | Down | | | | |
| **miR-200c-3p** | -0.5 | N/A | Down | **miR-3591** | 1.8 | N/A | Up | | | | |
| **miR-201-5p** | -0.6 | N/A | Down | **miR-3596a** | -0.7 | N/A | Down | | | | |
| **miR-203** | N/A | 0.8^ | Up | **miR-6314** | -0.5 | N/A | Down | | | | |
| **miR-206-3p** | 2.8 | N/A | Up | **miR-6319** | -0.7 | N/A | Down | | | | |
| **miR-207** | 2.1 | N/A | Up | **miR-6321** | -0.5 | N/A | Down | | | | |
| **miR-216a-5p** | -0.6 | N/A | Down | **miR-6333** | -0.6 | N/A | Down | | | | |
| **miR-217-3p** | -0.4 | N/A | Down | | | | | | | | |
